# Two new species of zooplanktivorous haplochromine cichlids from Lake Victoria, Tanzania

**DOI:** 10.3897/zookeys.256.3871

**Published:** 2013-01-02

**Authors:** Marnix P. de Zeeuw, Irene Westbroek, Martien J.P. van Oijen, Frans Witte

**Affiliations:** 1 Naturalis Biodiversity Center, The Netherlands; 2 Institute of Biology Leiden, Leiden University, The Netherlands

**Keywords:** Allopatric populations, Cichlidae, colour polymorphism, East Africa, endangered species, *Haplochromis*, zooplanktivores

## Abstract

Two new species of zooplanktivorous haplochromine cichlids from Lake Victoria, Tanzania, are described and illustrated. These species closely resemble each other. Their affinities to other zooplanktivorous haplochromines from Lake Victoria are discussed. *Haplochromis argens* **sp. n.**, which featured under nicknames (mainly *Haplochromis* “argens”) in more than 50 papers, was caught both in the Mwanza Gulf and the Emin Pasha Gulf, whereas *Haplochromis goldschmidti* **sp. n.** was only found in the Emin Pasha Gulf. Of the latter species only males are available, but it seems unlikely that it represents a case of male colour polymorphism as several presumably unrelated characters differ in sympatry between the two species, suggesting that there is no gene flow. Statistical analysis revealed that the overall difference between the two species is greater than that between the populations from the two locations. Body depth of the two species in sympatry in the Emin Pasha Gulf was more similar than that of *Haplochromis goldschmidti* **sp. n.** and the allopatric population of *Haplochromis argens* **sp. n.** from the Mwanza Gulf,which mayindicate an overall environmental effect. However, several measurements related to the width of snout and mouth differed more between the populations of the two species in sympatry than between the allopatric populations. In contrast to a group of zooplanktivorous species that recovered successfully after environmental changes in the lake, *Haplochromis argens* **sp. n.** is among a group that became extremely rare and probably is in danger of extinction; the conservation status of *Haplochromis goldschmidti* **sp. n.** is currently unknown.

## Introduction

In this paper two zooplanktivorous haplochromine species from Lake Victoria are described. One of these species, nicknamed *Haplochromis* “argens,” was common in the Mwanza Gulf until 1985 and also caught in other areas in the Tanzanian part of the lake. Since the late 1970s, this species has been the subject of studies on ecology (e.g. Witte 1984b, Goldschmidt et al. 1990), morphology (e.g. van der Meer and Bowmaker 1995, van der Meer et al. 1995), behavioral biology (Smith and Wooton 1994) and physiology (Rosenberger and Chapman 2000, Melnychuk and Chapman 2002). The second species, which closely resembles *Haplochromis* “argens,” was nicknamed *Haplochromis* “dusky argens”. It was only caught in the Emin Pasha Gulf in 1985 and 1986, concomitantly with *Haplochromis* “argens” (Goldschmidt and de Visser 1990).

Up to the 1980s, the zooplanktivorous haplochromines were, both in number and biomass, the second most abundant group of demersal fishes in sub-littoral areas of the Mwanza Gulf. A one hour tow of a bottom otter trawl (head rope 25 m) contained on average 1140 kg of haplochromines, of which 27% (more than 100,000 individuals) were zooplanktivores (Goldschmidt et al. 1993, Witte et al. 2012b). During the 1980s it became clear that the ecosystem of Lake Victoria was subject to a perturbation of an enormous magnitude. The population of Nile perch, *Lates niloticus* (Linnaeus 1758), an introduced predator, boomed (Ogutu-Ohwayo 1990, Goudswaard et al. 2008) and concomitantly widespread eutrophication became apparent (e.g. Kaufman 1992; Witte et al. 1992a; Hecky 1993; Hecky et al. 1994, 2010; Seehausen et al. 1997a; Wanink et al. 2001). As a result, the haplochromine cichlids in the sub-littoral waters of Lake Victoria almost vanished (Ogutu-Ohwayo 1990; Witte et al. 1992b).

From 1987 to 1992, haplochromines were extremely rare in trawl catches in the Mwanza Gulf; however, with the subsequent decline of Nile perch due to heavy fishing, a slow recovery of some zooplanktivorous species was noticed (Seehausen et al. 1997b; Witte et al. 2000, 2007a, b). This resurgence is continuing till the present day. In 2005 the zooplanktivorous haplochromines, which used to be second in importance after the detritivorous haplochromines, were the dominant trophic group and even more abundant than before the environmental changes (Witte et al. 2007a). Initially, *Haplochromis* “argens” was not present among the resurgent zooplanktivores, but the species has been caught again since 2002, albeit in very low numbers (Wanink JH, Kishe-Machumu M and Witte F, unpublished data).

As *Haplochromis* “argens” has already made its appearance in more than 50 articles, is bred in captivity by scientists and hobbyists, and currently is caught again in the lake, a formal taxonomic description is urgently needed and presented in this paper.

In the Emin Pasha Gulf, males of *Haplochromis* “argens” and males that resembled this species were caught in the same hauls. The latter males differed from *Haplochromis* “argens” in aspects of their overall nuptial body colouration, the colour of their caudal fin, and number and position of the egg spots on the anal fin (Goldschmidt and de Visser 1990). These males were referred to as *Haplochromis* “dusky argens” by Goldschmidt and de Visser (1990). The taxonomic status of these specimens is investigated, and they are described as a new species in this paper.

## Material and methods

**Figure 1.**
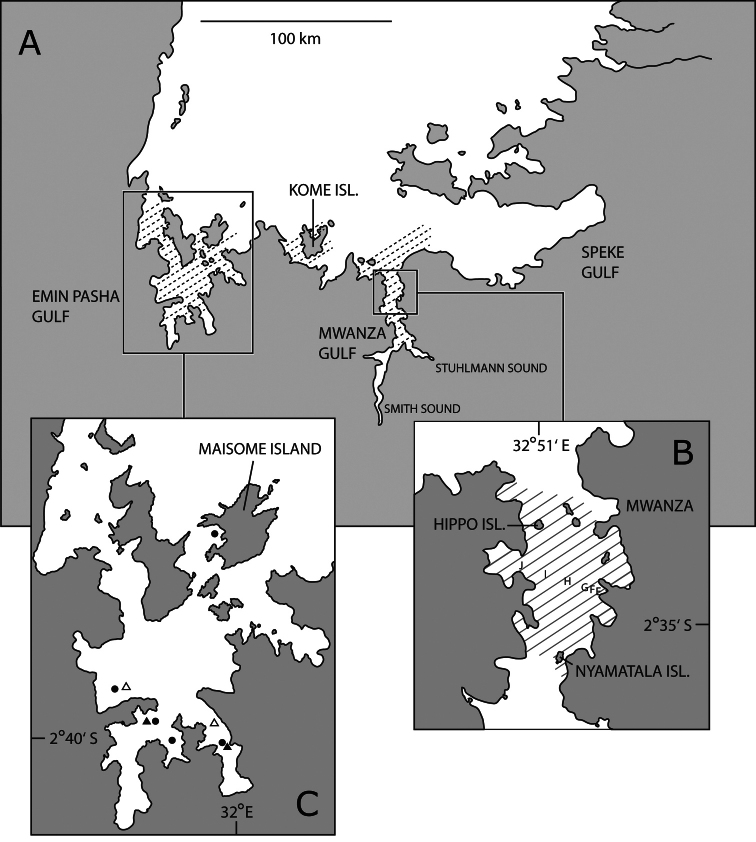
Tanzanian part of Lake Victoria. **A**, distribution of *Haplochromis argens* sp. n.(hatched area) **B** Mwanza Gulf with catch localities of type specimens of *Haplochromis argens* sp. n.(hatched area); E to J represent stations along the research transect where data on abundance of *Haplochromis argens* sp. n. were collected **C** Emin Pasha Gulf with catch localities of type specimens of *Haplochromis argens* sp. n. (filled circles) and type specimens of *Haplochromis goldschmidti* sp. n. (filledtriangles); open triangles indicate other catch localities of *Haplochromis goldschmidti* sp. n.

The type specimens of both species were collected in the Tanzanian part of Lake Victoria. All type material was collected between May 1975 and August 1986, before the collapse of the haplochromines in the Tanzanian part of the lake due to the Nile perch upsurge and eutrophication (e.g. Witte et al. 1992a, b, 2012a; Seehausen et al. 1997a; Wanink et al. 2001; Goudswaard et al. 2008). *Haplochromis* “argens” was collected in the Mwanza Gulf (between Nyamatala Island and Hippo Island, depth range about 6–16 m) and in the Emin Pasha Gulf (Fig. 1), mainly with bottom trawls, occasionally with gill nets. The type material of *Haplochromis* “argens” comprises 77 specimens (size range of 53.1–77.4 mm SL): 36 males and eight females from the Mwanza Gulf and 33 males and no females from the Emin Pasha Gulf. The type specimens (51 males; size range 50.7–69.2 mm SL) of *Haplochromis* “dusky argens” were collected in the Emin Pasa Gulf with bottom trawls on 22 and 23 July 1985, at two localities (depth range 4–8 m; Fig. 1). The live colours of some individuals were photographed in a small perspex tank filled with water (Barel et al. 1977). Immediately after they were caught, the fish were stored on ice. In the laboratory they were preserved in 10% formalin neutralized with borax. After several months to several years the fishes were sent to the Netherlands, where they were rinsed with tap water and stepwise transferred to 70% ethanol.

Ten male specimens of *Haplochromis* “argens” were dissected. The oral jaws were removed from nine specimens and the pharyngeal jaws from four specimens. From six specimens the first gill arch was dissected. Of *Haplochromis* “dusky argens,” six specimens were dissected. From five specimens the oral jaws and the intestines were removed and from three specimens the pharyngeal jaws and the first gill arch. The description of the shape of the oral and pharyngeal jaws and their dentition was based on the dissected elements, as were the counts of the gill filaments.

Linear measurements and counts were collected from 57 specimens of *Haplochromis* “argens” and 19 specimens of *Haplochromis* “dusky argens.” Counts included numbers of scales, teeth, gill rakers and vertebrae, the latter obtained from radiographs. Additional specimens for which counts and measurements were not made are also designated as type specimens; these include specimens used for colour descriptions (from colour slides) and other qualitative characters, specimens used for dissection, and specimens from which tissue samples for DNA analysis had been taken. Terminology and measurements follow Barel et al. (1977), Hoogerhoud and Witte (1981) and Witte and Witte-Maas (1981). Abbreviations used in the text are explained in Table 1.

**Table 1.** Measurements of *Haplochromis argens*, proportional to standard length or head length. Means and standard deviations were calculated over all measured type specimens, including the holotype.

		**Holotype**	**Paratypes (n = 56)**	**Mean ± SD (n = 57)**
standard length SL (mm)		67.3	53.1–75.5	63.1 ± 5.4
body depth (BD)	%SL	28.8	26.0–30.7	28.2 ± 1.1
pectoral fin length (PFL)	%SL	27.3	25.4–30.9	27.8 ± 1.2
caudal peduncle length (CPL)	%SL	21.7	18.5–23.5	20.7 ± 1.1
caudal peduncle depth (CPD)	%SL	9.7	9.1–11.1	10.0 ± 0.5
caudal fin length (CFL)	%SL	22.7	20.1–25.5	23.9 ± 1.0
head length (HL)	%SL	31.7	31.0–35.6	32.9 ± 0.9
snout length (SnL)	%HL	26.3	23.6–29.7	26.6 ± 1.6
snout width (SnW)	%HL	27.6	20.6–27.8	24.5 ± 1.7
head width (HW)	%HL	41.3	32.0–43.8	40.0 ± 1.8
interorbital width (IOW)	%HL	22.1	18.8–23.8	20.8 ± 1.1
preorbital width (POW)	%HL	24.4	20.6–25.7	23.4 ± 1.2
lachrymal width (LaW)	%HL	21.6	17.3–23.2	20.2 ± 1.4
preorbital depth (POD)	%HL	15.5	13.1–18.7	15.3 ± 1.2
eye length (EyL)	%HL	36.6	30.9–39.0	35.7 ± 2.1
eye depth (EyD)	%HL	33.4	27.4–35.2	31.6 ± 1.7
cheek depth (ChD)	%HL	16.9	11.9–20.5	16.7 ± 1.7
lower jaw length (LJL)	%HL	40.4	37.6–44.3	40.5 ± 1.5
lower jaw width (LJW)	%HL	13.6	11.0–15.6	13.2 ± 1.0
EyD/EyL		0.9	0.8–1.0	0.9 ± 0.04
LJL/LJW		3.0	2.6–3.8	3.1 ± 0.27

Measurements were taken to the nearest 0.1 mm using digital callipers with needles glued to the ends. For comparison with earlier described haplochromine species from Lake Victoria (e.g. Greenwood 1981) we transformed the measurements to proportions (percentages) of standard length (SL) or head length (HL). We present the proportional measurements to the nearest 0.1%, but it should be noted that they may deviate ± 0.2% because measurements were only accurate to the nearest 0.1 mm; the same is true for measurements presented in other species descriptions of Lake Victoria haplochromines. Unless stated otherwise, qualitative characters described as being relatively small or large refer to size relative to that of *Haplochromis (Astatotilapia) elegans* Trewavas, 1933. Morphologically, this insectivore from Lake George is a modal haplochromine cichlid. Its skeletal elements are described in Barel et al. (1976).

A comparison was made between *Haplochromis* “dusky argens” and the two populations of *Haplochromis* “argens” from the Mwanza Gulf and the Emin Pasha Gulf. As no females are available for *Haplochromis* “dusky argens,” we only compared males. To test for differences in linear measurements of the three populations in general, MANCOVA was used. ANCOVAs were used to identify differences among the three populations in specific linear measurements. Data were log-*e* transformed to achieve linearity. The factors “Species” (two species) and “Location” (two locations) were investigated, and log standard length (SL) was used as covariate. Parameter estimates derived from the GLM (analysis of covariance) procedure were used to define and plot the power function between SL and individual taxonomic measurements and to calculate the relative differences in individual measurements between *Haplochromis* “dusky argens” and the two populations of *Haplochromis* “argens”. For testing of interactions and main effects, Sum of Squares Type II was used. Whether the residuals followed a normal distribution was investigated with the Kolmogorov-Smirnov Test. For statistical analysis of the morphometric data, SPSS version 14.0 for Windows was used.

Live (juvenile) *Haplochromis (Psammochromis) cassius* Greenwood & Barel, 1978, are similar to *Haplochromis* “argens”. To compare *Haplochromis* “argens” with *Haplochromis cassius* which was described from only five females (some of them much larger than *Haplochromis* “argens”), we used measurements from six specimens of *Haplochromis cassius*: three specimens from the collection of Naturalis Biodiversity Center (RMNH.PISC.63199, RMNH.PISC.63200 and RMNH.PISC.74187) and three specimens from the collection of the Natural History Museum, London (BMNH 1987.2.4.1, BMNH 1987.2.4.2 and BMNH 1987.2.4.5). These specimens have a size range (67.4 – 79.3 mm SL) comparable to that of *Haplochromis* “argens”.

To describe changes in abundance of *Haplochromis* “argens” during the period 1979 to 2011, we compared the frequency of occurrence (= percentage of catches containing one or more individuals of *Haplochromis* “argens”) and the average numbers of individuals that were caught in trawl tows of 10 minutes duration with a small boat (20 or 25 hp) on a research transect in the Mwanza Gulf (Fig. 1; Witte et al. 1992). A bottom otter trawl (head rope 4.6 m, codend mesh 5 or 15 mm) was used during the day and a surface trawl (beam 4.5 m, codend mesh 5 mm) during the night.

Specimens referred to in this study are deposited in the Naturalis Biodiversity Center, Leiden (RMNH), the American Museum of Natural History, New York (AMNH), the Natural History Museum, London (BMNH) and the National Museum of Nature and Science, Tsukuba (NSMT), Japan.

## Results

### Species descriptions

#### Haplochromis argens

de Zeeuw, Westbroek & Witte sp. n.

urn:lsid:zoobank.org:act:B0288AFB-9C11-4D81-9B7B-E551951A7B2D

http://species-id.net/wiki/Haplochromis_argens

**Figure 2.**
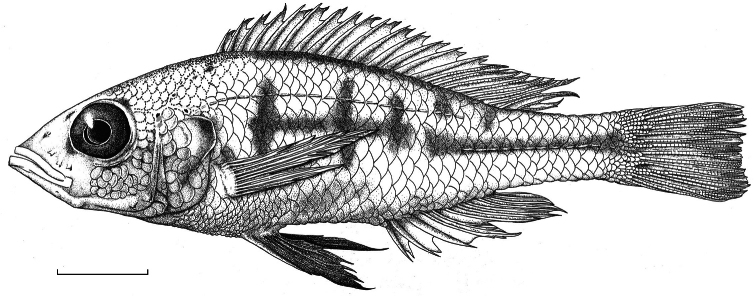
Habitus of *Haplochromis argens* sp. n. ♂ (holotype, RMNH.PISC.83588). Scale bar equals 10 mm. Drawing by I. Westbroek, missing (= dotted) scales added by M. van Oijen.

Figs 2 [Fig F3] [Fig F4] –5 , Tables 1 , 2

##### Cheironyms used.

Haplochromis argens: Goldschmidt 1994: 179, 199–200; *Haplochromis argens*; Verheyen et al. 1989: 93, 94, 96; van der Meer 1995: 26, 30–32; van der Meer and Bowmaker 1995: 232–239; Goldschmidt 1996: 164, 184–185; Hilder and Pankhurst 2003: 194; Collin et al. 2004: 769; Carleton et al. 2008.

*Haplochromis* “argens”; Zihler 1982: 568; Barel 1983: 384, 385, 407, 418; Witte 1984a: 604, 611; Witte 1984b: 159–161, 163; Barel 1985: chapter 1, 384, 385, 407, 418; Gottfried 1986: 1029; Witte 1987, chapter 1: 611, chapter 2: 67, 76, chapter 3: 8–13, 19; Goldschmidt 1989a: 24, 27, 29–39, 41, 42, 44, 45, 53, 55, 58, 59, 61–63, 65–69, 71–75, 77, 82, 84, 86–89, 97–101, 103, 118, 119, 148, 158, 160, 162, 166; Goldschmidt 1989b: 122–126, 129–131; Wanink et al. 1989: 25; van der Meer 1989: 52, 53; Goldschmidt and de Visser 1990: 129, 130, 132; Goldschmidt et al. 1990: 344, 346–351, 353; Goldschmidt and Witte 1990: 356–367; Barel et al. 1991: 262; Goldschmidt 1991: 181, 182, 185, 187; van der Meer 1991a: 91–94, 96; van der Meer 1991b: 3, 5, 6, 9–12, 14–22, 24–26, 28, 68, 76, 77, 83, 85–87, 89–93, 99, 101, 104; Goldschmidt and Witte 1992: 104; Kaufman 1992: 848, 849; Witte et al. 1992b: 11, 13, 17, 25, 27, 28; Barel 1993: 366; Smith and Wootton 1994: 99–103; Balshine-Earn 1995: 5; Seehausen 1995: 143, 146; Seehausen and Witte 1995: 101; van der Meer et al. 1995: 116–129; Smith and Wootton 1995: 12; Anker and Dullemeijer 1996: 4–11; de Visser and Barel 1996: 4; Seehausen 1996: 51, 55, 255, 256; Smit and Anker 1997: 9–17; Seehausen et al. 1997b: 899; Witte et al. 1997: 591; Wanink 1998: 159, 232; Rinkes 1999: 46, 47, 54, 56–58, 60, 62, 76, 77, 79, 97; de Visser 2000: 18; Tacon et al. 2000: 63; Witte and Wanink 2000: 78–81, 84; Seehausen et al. 2003: 272; Witte et al. 2005: 320; Kishe-Machumu 2012: 35; van Rijssel and Witte 2012.

H. “Argens”; Kaufman and Seehausen 1995: 148.

*Haplochromis* *(?)* “argens”; Wanink and Witte 2000: 563; Witte et al. 2000: 234, 235, 237; Witte et al. 2003: 108; Niemantsverdriet 2005: 151, 155; Witte et al. 2007a: 1153.

*Haplochromis* sp. “argens”; Mizoiri et al. 2008: 228, 241, 254.

*Yssichromis argens*; van Staaden et al. 1995: 168; Chapman et al. 1995: 1277, 1279–1282, 1285; Huber et al. 1997: 170; Rosenberger and Chapman 2000: 498; Melnychuk and Chapman 2002: 107.

*Yssichromis* sp. “argens”; Mizoiri et al. 2008: 228.

**Type-locality.** Tanzania, Lake Victoria, Mwanza Gulf (ca 2°29'–2°36'S; 32°48'–32°54'E) and Emin Pasha Gulf (ca 2°18'–2°41'S; 31°47'–31°59' E).

**Holotype.** RMNH.PISC.83588^4^, ♂, 67.3 mm SL, Tanzania, Lake Victoria, Mwanza Gulf, 8.iii.1979, HEST.

**Paratypes.** All type specimens collected by *Haplochromis* Ecology Survey Team (HEST) in Mwanza Gulf, Tanzania, Lake Victoria, except where noted otherwise. Size of specimens given as standard length. RMNH.PISC.72831^5^, ♀, 71.0 mm, 30.v.1980; RMNH.PISC.72884^5, 6^, ♂, 74.4 mm, 31.v.1980; RMNH.PISC.73097, ♀, 71.3 mm, 27.ix.1977; RMNH.PISC.81202^5^, ♀, 65.6 mm, 11.v.1978; RMNH.PISC.83587, ♂, 77.4 mm, 31.v.1975; RMNH.PISC.83589^4^, ♀, 75.5 mm, 21.iv.1980; RMNH.PISC.83590^4^, ♂, 68.2 mm, 22.iv.1980; RMNH.PISC.83606^4^, ♂, 73.7 mm, 22.iv.1980; RMNH.PISC.83607^4^, ♂, 65.7 mm, 15.iv.1980; RMNH.PISC.83608^5^, ♂, 65.7 mm, 31.v.1975; RMNH.PISC.83609^4^, ♂, 67.4 mm, 22.iv.1980; RMNH.PISC.83610^4^, ♂, 66.2 mm, 30.ix.1977; RMNH.PISC.83611^4^, ♂, 69.5 mm, 30.ix.1977; RMNH.PISC.83612^4^, ♀, 71.1 mm, 30.ix.1977; RMNH.PISC.83613^4^, ♀, 68.1 mm, 30.ix.1977; RMNH.PISC.83614^4^, ♀, 71.9 mm, 8.ix.1977; RMNH.PISC.83615^4^, ♂, 57.7 mm, 8.ix.1977; RMNH.PISC.83616^4^, ♂, 70.2 mm, 19.viii.1977; RMNH.PISC.83617^4^, ♀, 71.5 mm, 19.viii.1977; RMNH.PISC.83618^4^, ♂, 66.1 mm, 7.ix.1977; RMNH.PISC.83619^4^, ♂, 66.5 mm, 21.xii.1977; RMNH.PISC.83620^4^, ♂, 65.8 mm, 10.x.1977; RMNH.PISC.83621^1^, ♂, circa 70 mm, 30.ix.1977; RMNH.PISC.83622^5^, ♂, 59.3 mm, 22.vi.1985, Emin Pasha Gulf; RMNH.PISC.83623^5^, ♂, 57.2 mm, 22.vi.1985, Emin Pasha Gulf; RMNH.PISC.83624^5^, ♂, 59.6 mm, 23.vi.1985, Emin Pasha Gulf; RMNH.PISC.83625^4^, ♂, 58.3 mm, 23.vi.1985, Emin Pasha Gulf; RMNH.PISC.83626^4^, ♂, 58.5 mm, 23.vi.1985, Emin Pasha Gulf; RMNH.PISC.83627^4^, ♂, 54.3 mm, 23.vi.1985, Emin Pasha Gulf; RMNH.PISC.83628^4^, ♂, 59.6 mm, 23.vi.1985, Emin Pasha Gulf; RMNH.PISC.83629^4^, ♂, 55.4 mm, 23.vi.1985, Emin Pasha Gulf; RMNH.PISC.83630^4^, ♂, 53.1 mm, 23.vi.1985, Emin Pasha Gulf; RMNH.PISC.83631^4^, ♂, 66.8 mm, 23.vi.1985, Emin Pasha Gulf; RMNH.PISC.83632^4^, ♂, 67.0 mm, 23.vi.1985, Emin Pasha Gulf; RMNH.PISC.83633^4^, ♂, 60.4 mm, 23.vi.1985, Emin Pasha Gulf; RMNH.PISC.83634^4^, ♂, 53.9 mm, 23.vi.1985, Emin Pasha Gulf; RMNH.PISC.83635^4^, ♂, 62.6 mm, 23.vi.1985, Emin Pasha Gulf; RMNH.PISC.83636^4^, ♂, 66.6 mm, 23.vi.1985, Emin Pasha Gulf; RMNH.PISC.83637^4^, ♂, 69.2 mm, 23.vi.1985, Emin Pasha Gulf; RMNH.PISC.83638^4^, ♂, 65.8 mm, 23.vi.1985, Emin Pasha Gulf; RMNH.PISC.83639^4^, ♂, 61.3 mm, 23.vi.1985, Emin Pasha Gulf; RMNH.PISC.83640^4^, ♂, 73.4 mm, 23.vi.1985, Emin Pasha Gulf; RMNH.PISC.83641^4^, ♂, 64.1 mm, 23.vi.1985, Emin Pasha Gulf; RMNH.PISC.83642^4^, ♂, 63.1 mm, 23.vi.1985, Emin Pasha Gulf; RMNH.PISC.83643^4^, ♂, 63.7 mm, 23.vi.1985, Emin Pasha Gulf; RMNH.PISC.83644^4^, ♂, 62.1 mm, 23.vi.1985, Emin Pasha Gulf; RMNH.PISC.83645^4^, ♂, 64.1 mm, 23.vi.1985, Emin Pasha Gulf; RMNH.PISC.83646^4^, ♂, 61.6 mm, 23.vi.1985, Emin Pasha Gulf; RMNH.PISC.83647^4^, ♂, 59.8 mm, 25.viii.1981; RMNH.PISC.83648^4^, ♂, 61.6 mm, 10.x.1977; RMNH.PISC.83649^4^, ♂, 61.5 mm, 21.xii.1977; RMNH.PISC.83650^4^, ♂, 57.7 mm, 6.iii.1979; RMNH.PISC.83651^4^, ♂, 60.6 mm, 13.vii.1979; RMNH.PISC.83652^4^, ♂, 58.0 mm, 1.ii.1979; RMNH.PISC.83653^4^, ♂, 53.5 mm, 18.xi.1981; RMNH.PISC.83655^4^, ♂, 61.8 mm, 14.v.1979; RMNH.PISC.83656^4^, ♂, 59.0 mm, 27.iii.1979; RMNH.PISC.83657^4^, ♂, 61.6 mm, 27.iii.1979; RMNH.PISC.83660^4^, ♂, 56.8 mm, 12.iii.1979; RMNH.PISC.83661^4^, ♂, 57.9 mm, 12.iii.1979; RMNH.PISC.83662^4^, ♂, 59.7 mm, 14.v.1979; RMNH.PISC.83663^4^, ♂, 58.6 mm, 14.v.1979; RMNH.PISC.83673^4^, ♂, 55.5 mm, 23.vi.1985, Emin Pasha Gulf; RMNH.PISC.83688^1–3^, ♂, 61.4 mm, 6.iii.1979; RMNH.PISC.83689^1–3^, ♂, 62.2 mm, 14.v.1979; RMNH.PISC.83694^1, 5^, ♂, 63.1 mm, 21.vi.1985, Emin Pasha Gulf; RMNH.PISC.83697^1, 5^, ♂, 59.1 mm, 23.vi.1985, Emin Pasha Gulf; RMNH.PISC.83698^1–3, 5^, ♂, 61.0 mm, 23.vi.1985, Emin Pasha Gulf; RMNH.PISC.83703^3, 6^, ♂, 66.4 mm, 23.vi.1985, Emin Pasha Gulf; RMNH.PISC.83704^1, 3, 6^, ♂, 68.4 mm, 23.vi.1985, Emin Pasha Gulf; RMNH.PISC.83705^1–3, 6^, ♂, 67.5 mm, 23.vi.1985, Emin Pasha Gulf; RMNH.PISC.83706^1, 5^, ♂, 68.8 mm, 27.vi.1985, Emin Pasha Gulf; RMNH.PISC.84067^5, 7^, ♂, 72.1 mm, 15.viii.1986; AMNH 255035^4^, ♂, 62.2 mm, 14.v.1979; BMNH 2012.1.5.2 ^4^, ♂, 62.8 mm, 3.v.1979; NSMT-P 106959^4^, ♂, 62.6 mm, 3.v.1979.

^1^ dissected to describe oral jaws; ^2^ dissected to describe pharyngeal jaws; ^3^ dissected to count gill filaments; ^4^ proportional measurements taken (Table 1); ^5^colour picture available; ^6^colour picture of anal fin available; ^7^ specimen of which Dr E. Verheijen, Royal Belgian Institute of Natural Sciences, has taken a tissue sample for DNA analysis.

##### Diagnosis.

Small sized (< 8 cm SL), slender (BD < 31% SL), micrognathic (LJL < 45% HL) zooplanktivorous *Haplochromis* species with slightly curved to straight dorsal head profile. Relatively long and slender, mainly bicuspid, teeth in oral jaws. Premaxillary dentigerous area extending almost to caudal end of dentigerous arm. Both males and females silvery with conspicuously ivory-white lips. Three to five, generally faint vertical stripes on flank; faint traces of a dark mid-lateral band occasionally present. Males with yellow to greenish sheen on flank.

##### Description.

Proportional measurements of type material given in Table 1.

**Habitus.** See Fig. 2. Body slender. Dorsal head profile straight to slightly curved, occasionally moderately curved. Premaxillary pedicel slightly prominent. Mouth oblique. Lips not thickened. Medial part of premaxilla slightly expanded. Caudal part of maxilla not bullate. Vertical through caudal tip of maxilla running through iris, just rostral to pupil. Lateral snout outline isognathous and obtuse, in larger specimens slightly prognathous. Jaws equal anteriorly or lower jaw slightly protruding. Mental prominence slightly pronounced. Retro-articular processes of right and left mandible touching each other, interrupting ventral body outline. Eye approximately circular and medium to large. Generally an aphakic aperture present in pupil. Cephalic lateral line pores not enlarged.

**Scales.** Cheek, gill cover and rostral part of dorsal head surface covered with cycloid scales. Nape and rostral part of dorsum with mixture of cycloid and weakly ctenoid scales. Chest with ctenoid, weakly ctenoid and some cycloid scales. Scales on remaining part of body mainly ctenoid. Scales on chest smaller than those on ventral and ventro-lateral part of body; size transition gradual. Small elongated scales on basal quarter to half of caudal fin. Three to seven (mode 6) scales between upper lateral line and dorsal-fin origin, four to eight (mode 6) between pectoral- and pelvic-fin bases.

**Fins.** Pelvic fins just reaching or slightly surpassing rostral-most point of anal-fin origin. Pelvic fins with first soft rays slightly produced in both sexes, in males occasionally filamentous. Caudal tip of anal fin not reaching caudal-fin origin. Caudal-fin outline truncate to slightly emarginate.

**Gill apparatus.** Description based on lateral gill rakers and lateral hemibranch of first gill arch. Number of gill rakers on lower part of gill arch 11–12. Lower two to three rakers reduced (= very short), next one to two short, followed by two to six slender and longer ones. Remaining rakers hooked, bifid, trifid or quadrifid. Rakers generally closely set, viz. touching each other over major part of length. Number of gill filaments 94 to 106.

**Viscera.** Ratio between intestine length and SL: 1.0–1.4 (n = 25).

**Figure 3. F3:**
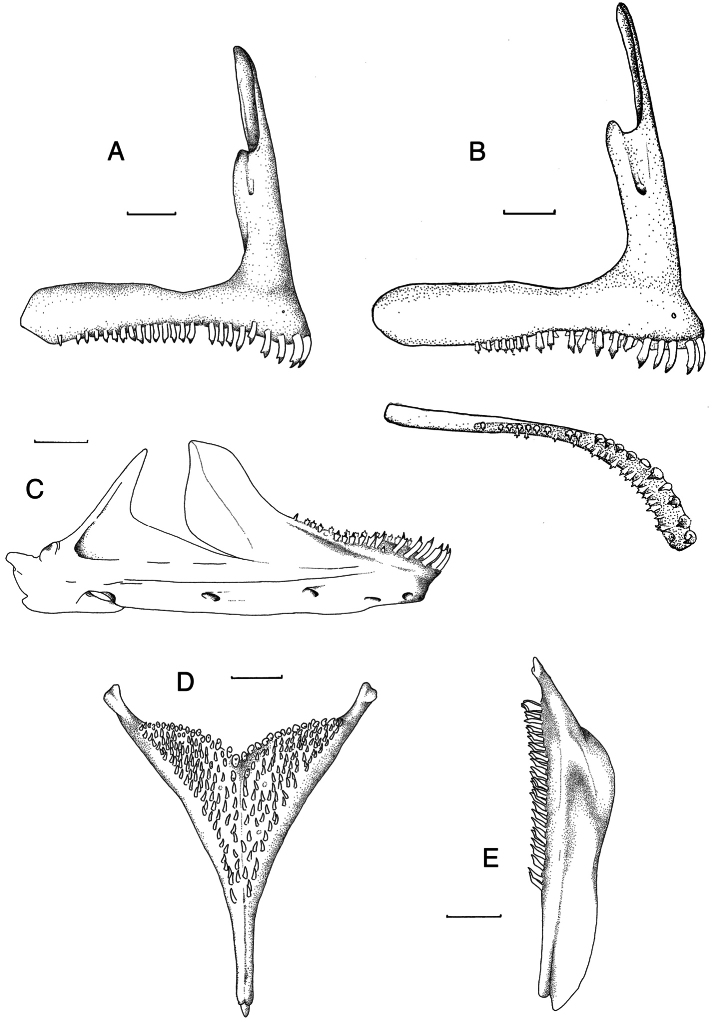
Skeletal elements of *Haplochromis argens* sp. n. **A** Right premaxilla, lateral view (RMNH.PISC.83705) **B** Right premaxilla, lateral (top) and occlusal (bottom) views (RMNH.PISC.83621), illustrating, for this species, a rare case of a posteriorly edentulous premaxilla **C** Right lower jaw, lateral view (RMNH.PISC.83697) **D** Lower pharyngeal element, dorsal view (RMNH.PISC. 83706) **E** Lower pharyngeal element, lateral view (RMNH.PISC.83706). Scale bars equal 1 mm. **A, C,** **D** drawn by I. Westbroek, **B** by M. van Oijen.

**Oral jaws.** (Fig. 3 A–C) Premaxillary ascending arm equal to or longer than dentigerous arm (asc./dent. arm ratio 1.0 to 1.1). Angle between the arms 77° to 81°. Symphyseal articulation facet not present. Lower jaw slightly more elongated than generalized type (length/height ratio 2.3 to 2.5). Upper half of dentary with distinct outwardly directed flare. Mental prominence slightly pronounced.

**Oral teeth shape.** (Fig. 3 A–C) Generally teeth in outer row of both premaxilla and lower jaw bicuspid or weakly bicuspid, with some unicuspid or tricuspid teeth interspersed. In specimens over 65 mm SL, weakly bicuspids and unicuspids may dominate. Major cusp of bicuspids isoscelene to subequilateral, protracted and acutely pointed. Flange generally absent, when present very small. Minor cusp weakly developed to distinct, relatively short compared to major cusp. Cusp gap wide. In labial view, neck slender to moderately slender, crown not or slightly expanded. In lateral view, crown compressed. Outer-row teeth in both premaxilla and lower jaw recurved. Inner rows in both jaws with mainly tricuspid or weakly tricuspid teeth.

**Oral teeth size.** Outer-row teeth relatively long and slender, gradually decreasing in size from rostral to caudal.

**Dental arcade and tooth band.** (Fig. 3B) Rostrally dental arcade rounded. Outer row generally occupying almost total length of dentigerous arm of premaxilla, in two specimens (RMNH.PISC.83697 and RMNH.PISC.83621; Fig. 3B) edentulous part about 25% of arm. Outer row in lower jaw not, or just, reaching coronoid wing in most dissected specimens. In one case caudal-most tooth relatively high on coronoid wing. One or two inner rows in rostral part of both jaws, decreasing to zero in caudal part.

**Teeth counts and setting.** Outer row of upper jaw (l+r premaxilla) with 30–52 teeth. In both jaws outer-row teeth regularly set, their placement wider rostrally than laterally.

**Tooth implantation.** Outer-row teeth of premaxilla rostrally erect. Inner-row teeth recumbent. Outer-row teeth of lower jaw slightly procumbent, inner-row teeth erect.

**Lower pharyngeal element.** (Fig. 3 D, E) Lower pharyngeal element relatively small and slender (length/width ratio 1.2–1.3). Dentigerous area slightly broader than long (length/width ratio = 0.7–0.9). Suture straight.

**Pharyngeal teeth** **counts.** Caudal-most transverse row with about 30–38 teeth, medial longitudinal rows with eight to 11 teeth.

**Pharyngeal teeth shape.** Teeth in caudal-most transverse row hooked, major cusp only slightly incurved, blunt to slightly acute. Other teeth bevelled or pronounced. All teeth relatively fine and slender, medial teeth not coarser than other teeth.

**Vertebrae.** Total number of vertebrae in 57 specimens: 30 (12), 31 (39) or 32 (6), comprising 13–14 abdominal and 16–19 caudal vertebrae.

**Figure 4. F4:**
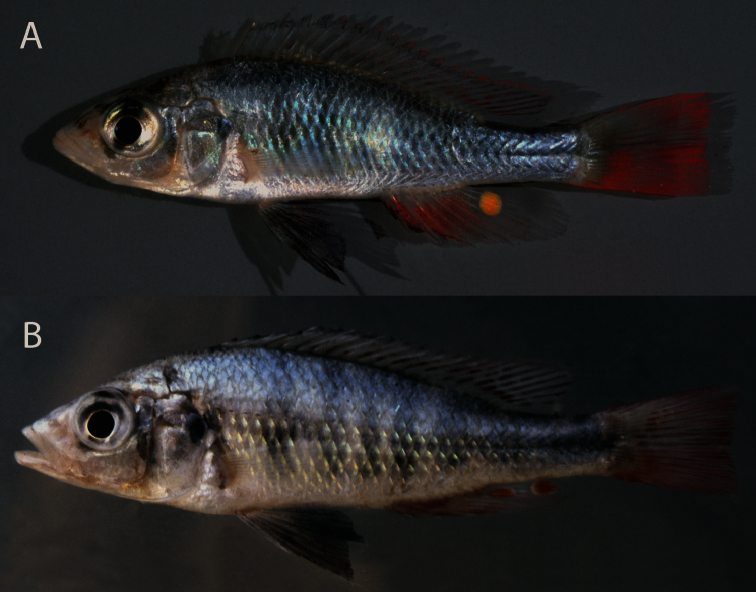
Live colours of *Haplochromis argens* sp. n. **A** sexually active ♂, 59.3 mm SL (paratype, RMNH.PISC.83622), Emin Pasha Gulf **B** sexually active ♂, 72.1 mm SL (paratype, RMNH.PISC.84067), Mwanza Gulf.

**Live colouration males.** (Fig. 4 A, B). Sexually active males with ivory to grey snout and cheek. Lips remarkably ivory-white with no or few pigment spots. Eye with grey outer ring and silver to golden inner ring. Lower jaw and interoperculum whitish. Gill cover silver, sometimes with grey to dusky flush. Dorsal head surface, dorsum and flank silvery-grey, dorsum with bluish to purplish sheen, flank with yellow to greenish sheen. Chest, belly and ventral side silvery-white.

Pelvic fins black; in specimens of Emin Pasha Gulf, medial side sometimes red. Anal fin rostrally faintly to distinctly red, rest of fin hyaline. One to two dark yellow to orange egg dummies with hyaline ring present on caudal part of anal fin. Caudal fin orange-red to wine-red. Dorsal fin hyaline with red streaks and spots. Lappets hyaline or reddish, rostral lappets sometimes dusky.

Dark grey to blackish markings: Nostril-, interorbital-, and supraorbital stripes, sometimes rather distinct. Lachrymal stripe distinct, but relatively short (i.e. small blotch at caudal end of lachrymal generally not reaching caudal tip of maxilla), sometimes extending over iris. Irregular preopercular vertical bar generally present. Opercular blotch distinct. Three to five, generally faint vertical stripes on flank. Traces of dark-grey mid-lateral band occasionally present.

**Live colouration females.** Live females basically with same colours as males, lacking bluish-purplish and yellow-greenish sheens and distinct red colouration in fins, but sometimes with faint red flush in caudal fin. In females upper lip usually with more pigment than in males. Of markings on head, only lachrymal stripe and opercular blotch distinct. Mid-lateral band sometimes more distinct than in males, vertical stripes faint.

**Figure 5.**
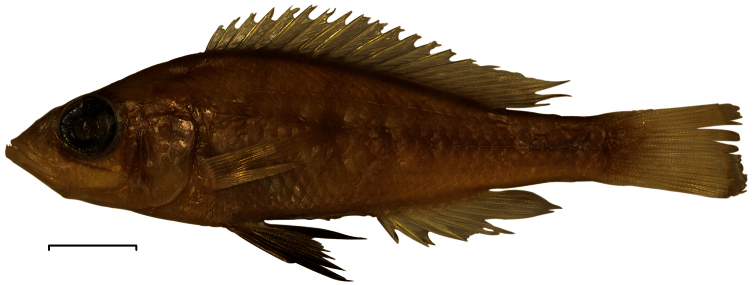
Preserved colours of *Haplochromis argens* sp. n. ♂ 67.3 mm SL (holotype, RMNH.PISC.83588). Scale bar equals 10 mm.

**Preserved colouration of males and females.** (Fig. 5) Body light brown, dorsally darker than ventrally. Snout, lips and lower jaw coloured as old ivory. Fins hyaline and light grey-brown in both sexes, except for black pelvic fins in adult males. Same markings, but slightly more distinct, as in live specimens.

##### Distribution.

*Haplochromis argens* is only known from the Tanzanian part of Lake Victoria. Specimens were caught in the Mwanza Gulf (from entrance of Stuhlmann Sound to entrance of gulf in north), in the south-western part of the Speke Gulf (near its entrance), in the area around Kome Island, and in the Emin Pasha Gulf (Fig. 1).

##### Habitat.

*Haplochromis argens* is a pelagic species from the littoral and sub-littoral zone. At night the species is virtually restricted to the two upper metres of the water column (Witte 1984b, Goldschmidt et al. 1990). By day, the highest densities were found at two to three metres from the surface, but individuals of this species were also caught with bottom trawls over sand and mud bottoms, and in gill nets and traps near rocks (Goldschmidt et al. 1990, Witte et al. 1992b).

##### Abundance.

During 1979–1982, *Haplochromis argens* was present in 70% of the bottom-trawl tows by day and in 100% of the surface trawl tows at night; the mean numbers of individuals per tow ranged from 6.2 to 18.3, respectively (Table 2). In 1987–1988, it occurred in 3% of the bottom trawl tows and the mean number of individuals per tow was 0.03; the species was absent in surface trawls. Thirty-four bottom-trawl tows in 1990–1999 captured no *Haplochromis argens*. From 2001 to 2011 more than 150 bottom-trawl tows contained about 15 individuals of *Haplochromis argens*, corresponding to a decline in catch per unit effort of more than 50 times compared to the 1979–1982 captures; no individuals were caught with surface trawls.

**Table 2.** Frequencies of occurrence (Foo) in trawl tows and mean numbers (± SD) per tow of *Haplochromis argens*. Tows of 10 minutes duration were made with bottom trawls (day) at stations E to J (depth range 7–14 m) and with surface trawls (night) at station G (14 m deep); in the period 1990-1999 no surface trawls were made; n indicates number of trawl tows.

**Station**	**Bottom trawl day**	**1979–1982^†^ n = 104**	**1987–1988^†^ n = 29**	**1990–1999^‡^ n = 34**	**2001–2011^§^ n > 150**
E–J	Foo	70 %	3 %	0 %	< 10 %
	Mean nr	6.2 ± 12.2	0.03 ± 0.19	0	< 0.1
	**Surface trawl night**	**1981/1982 n = 8**	**1987-1988^8|^ n = 26**	**-**	**2001-2011^1§^ n = 15**
G	Foo	100 %	0 %	-	27 %
	Mean nr	18.3 ± 12.4	0	-	0.5 ± 0.9

^†^ Data from Witte et al. (1992b); ^‡^ data from Witte et al. (2000); ^§^unpublished data from J.H Wanink; M.A. Kishe-Machumu, J.C. van Rijssel and F. Witte; ^|^ data from Wanink and Witte (1998).

**Food.** Before the ecological changes in Lake Victoria,the diet of *Haplochromis argens* comprised mainly zooplankton during the day; *Chaoborus* larvae were important at night (Goldschmidt et al. 1990). The current diet is unknown, but all the studied resurgent species in the Mwanza Gulf changed their diet (e.g. van Oijen and Witte 1996, Katunzi et al. 2003, Kishe-Machumu et al. 2008).

**Breeding.** *Haplochromis argens* is a female mouth brooder. Spawning sites are located at depths < 9 m (Goldschmidt and Witte 1990).

##### Etymology.

In reference to the silver male colouration, *Haplochromis argens* was given the nickname “argens” under the false assumption it was Latinized Greek for silver. Since this species is well known under its cheironym, we think it is best to upgrade the nickname to the species’ epithethon.

##### Comparisons.

The zooplanktivorous species *Haplochromis (Yssichromis) laparogramma* Greenwood & Gee, 1969, *Haplochromis (Yssichromis) pyrrhocephalus* Witte & Witte-Maas, 1987 and *Haplochromis (Yssichromis) heusinkveldi*, Witte & Witte-Maas, 1987, have shorter bicuspid teeth in the oral jaws than *Haplochromis argens*, and generally the premaxillary dentigerous arm is edentulous over the caudal ^1^/_4_ -^1^/_3_ versus the dentigerous portion extending almost to the caudal end of the dentigerous arm. The dental features of the zooplanktivorous/insectivorous *Haplochromis tanaos* van Oijen & Witte, 1996 and *Haplochromis thereuterion* van Oijen & Witte, 1996, are more or less similar to those of *Haplochromis argens*, but the former two species have more unicuspids. *Haplochromis argens* is *f*urther distinguished from these and other species by its colouration. Sexually active males of *Haplochromis tanaos* are dark blue, the females silvery with a distinct mid-lateral band and slightly less distinct dorso-lateral band. Sexually active males of *Haplochromis thereuterion* are black, the females coloured like females of *Haplochromis tanaos* (van Oijen and Witte 1996). The body of *Haplochromis argens* is less slender (BD 26.0–30.7% of SL, mean 28.2%, Table 1) than that of *Haplochromis tanaos* and *Haplochromis thereuterion* (22.1–27.1% and 24.4–27.6% of SL, respectively; Tables 3 and 7 in van Oijen and Witte 1996). Live *Haplochromis argens* is similar to (juvenile) *Haplochromis cassius* in colouration and general habitus. However, *Haplochromis cassius* has a broad, well defined mid-lateral band (Greenwood and Barel 1978), more and distinctly longer unicuspid teeth, more curved and more widely set teeth, and thicker lips than *Haplochromis argens*. The maximum size of *Haplochromis cassius* (99.0 mm SL) is larger than that of *Haplochromis argens*, but when comparing similar size ranges (see material and methods) *Haplochromis argens* has: a smaller head (ratio HL/SL: 31.0 – 35.6%, mean 32.9% versus 35.0 – 36.0%, mean 35.5% in *Haplochromis cassius*); a shorter snout (ratio SnL/HL: 23.6 – 29.7%, mean 26.6% versus 29.8 – 32.6%, mean 31.2% in *Haplochromis cassius*); larger eyes (ratio EyL/HL: 30.9 – 39.0%, mean 35.7% versus 26.8 – 28.8%, mean 28.3% in *Haplochromis cassius*). For comparison with *Haplochromis goldschmidti* sp. n., see below.

#### Haplochromis goldschmidti

Witte, Westbroek & de Zeeuw sp. n.

urn:lsid:zoobank.org:act:06762084-8C0C-49CC-A48D-065244CF8457

http://species-id.net/wiki/Haplochromis_goldschmidti

**Figure 6.**
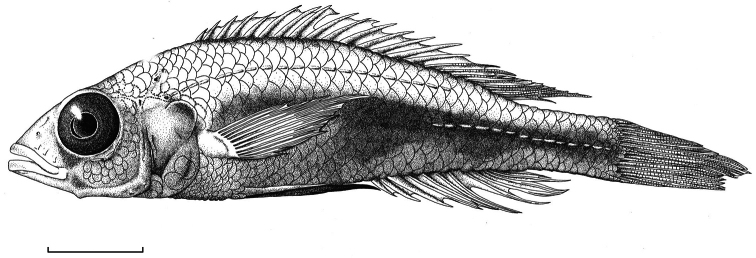
Habitus of *Haplochromis goldschmidti* sp. n. ♂ (holotype, RMNH.PISC.83573). Scale bar equals 10 mm. Drawing by I. Westbroek, missing (= dotted) scales added by M. van Oijen.

Figs 6 [Fig F7] [Fig F8] –9 , Table 3

##### Cheironyms used.

*Haplochromis* “dusky argens”; Goldschmidt 1989a: 148, 158, 160, 162, 166; Goldschmidt and de Visser 1990: 129, 130, 132; Goldschmidt 1991: 182, 187; van der Meer 1991b: 9–11, 15, 16, 19–22; van der Meer et al. 1995: 116, 117, 122–124, 126, 127.

##### Type-locality.

Tanzania, Lake Victoria, Emin Pasha Gulf (ca 2°35'–2°41'S; 31°47'–31°59'E).

##### Holotype.

RMNH.PISC.83573^3^, ♂, 60.3 mm SL, 23.vi.1985, HEST.

##### Paratypes.

Collected by the *Haplochromis* Ecology Survey Team (HEST). Size of specimens given as standard length. RMNH.PISC.80480^4^, ♂, 53.6 mm, 23.vi.1985; RMNH.PISC.80481, ♂, 56.8 mm, 23.vi.1985; RMNH.PISC.80482^4^, ♂, 63.4 mm, 23.vi.1985; RMNH.PISC.80483^4^, ♂, 52.9 mm, 23.vi.1985; RMNH.PISC.80484^4^, ♂, 65.3 mm, 23.vi.1985; RMNH.PISC.80485^4^, ♂, 69.2 mm, 23.vi.1985; RMNH.PISC.80493^4^, ♂, 50.7 mm, 23.vi.1985; RMNH.PISC.80494^4^, ♂, 52.2 mm, 23.vi.1985; RMNH.PISC.80495^5^, ♂, 58.7 mm, 23.vi.1985; RMNH.PISC.80496^5^, ♂, 57.4 mm, 23.vi.1985; RMNH.PISC.80497^5^, ♂, 55.0 mm, 23.vi.1985; RMNH.PISC.80498^5^, ♂, 60.4 mm, 23.vi.1985; RMNH.PISC.80499^5^, ♂, 67.4 mm, 23.vi.1985; RMNH.PISC.80501^5^, ♂, 61.1 mm, 23.vi.1985; RMNH.PISC.80502^5^, ♂, 62.6 mm, 23.vi.1985; RMNH.PISC.80503^5^, ♂, 57.7 mm, 23.vi.1985; RMNH.PISC.80507^5^, ♂, 52.8 mm, 23.vi.1985; RMNH.PISC.80508^5^, ♂, 62.9 mm, 23.vi.1985; RMNH.PISC.80512^5^, ♂, 59.3 mm, 23.vi.1985; RMNH.PISC.80513^5^, ♂, 63.6 mm, 23.vi.1985; RMNH.PISC.80514^5^, ♂, 61.7 mm, 23.vi.1985; RMNH.PISC.83574^3^, ♂, 62.6 mm, 23.vi.1985; RMNH.PISC.83575^4^, ♂, 57.5 mm, 23.vi.1985; RMNH.PISC.83576, ♂, 58.2 mm, 23.vi.1985; RMNH.PISC.83577^3^, ♂, 62.5 mm, 23.vi.1985; RMNH.PISC.83578^3^, ♂, 69.2 mm, 23.vi.1985; RMNH.PISC.83579^3^, ♂, 64.9 mm, 23.vi.1985; RMNH.PISC.83580^3^, ♂, 62.9 mm, 23.vi.1985; RMNH.PISC.83581^3^, ♂, 51.7 mm, 23.vi.1985; RMNH.PISC.83582^3^, ♂, 52.4 mm, 23.vi.1985; RMNH.PISC.83583^3^, ♂, 57.7 mm, 23.vi.1985; RMNH.PISC.83584^3^, ♂, 55.4 mm, 23.vi.1985; RMNH.PISC.83585^3^, ♂, 61.9 mm, 23.vi.1985; RMNH.PISC.83586^3^, ♂, 65.4 mm, 23.vi.1985; RMNH.PISC.83664^3^, ♂, 56.2 mm, 23.vi.1985; RMNH.PISC.83665^3^, ♂, 63.2 mm, 23.vi.1985; RMNH.PISC.83666^3^, ♂, 65.5 mm, 23.vi.1985; RMNH.PISC.83667^3^, ♂, 60.7 mm, 23.vi.1985; RMNH.PISC.83668^3^, ♂, 64.9 mm, 23.vi.1985; RMNH.PISC.83669^3^, ♂, 65.8 mm, 23.vi.1985; RMNH.PISC.83670^3^, ♂, 60.0 mm, 23.vi.1985; RMNH.PISC.83695^1, 2, 4^, ♂, 56.2 mm, 23.vi.1985; RMNH.PISC.83696^1, 4^, ♂, 58.0 mm, 23.vi.1985; RMNH.PISC.83699^1, 5^, ♂, 61.5 mm, 23.vi.1985; RMNH.PISC.83700^1, 5^, ♂, 55.3 mm, 23.vi.1985; RMNH.PISC.83701^2, 5^, ♂, 61.6 mm, 23.vi.1985; RMNH.PISC.83702^1, 2, 5^, ♂, 61.1 mm, 23.vi.1985; AMNH 255036^5^, ♂, 61.4 mm, 23.vi.1985; BMNH 2012.1.5.3^4^, ♂, 61.6 mm, 22.vi.1985; NSMT-P 106960^4^, ♂, 64.8 mm, 22.vi.1985.

^1^ dissected to describe the oral jaws and contents of stomachs and intestines; ^2^ dissected to describe pharyngeal jaws and to count gill filaments; ^3^ proportional measurements taken; ^4^ colour picture available; ^5^ colour picture of anal fin available.

##### Diagnosis.

Small sized (< 7 cm SL), slender (BD < 28% SL), micrognathic, zooplanktivorous *Hapolochromis* species (LJL < 45% HL in all but one specimens) with a slightly curved to straight dorsal head profile. Mainly bicuspid teeth in oral jaws. Generally premaxillary dentigerous arm edentulous over caudal ^1^/_5_ - ^1^/_4_. Males silvery with dusky flush on chest, flank, ventral side and ventral half of caudal peduncle.

##### Description.

Proportional measurements of type material provided in Table 3.

**Table 3.** Measurements of *Haplochromis goldschmidti* sp. n., proportional to standard length or head length. Means and standard deviations were calculated over all measured type specimens, including the holotype.

		**Holotype**	**Paratypes (n = 18) (n = 18)**	**Mean ± SD (n = 19) (n = 19)**
SL (mm)		60.3	51.7–69.2	61.2 ± 4.6
BD	%SL	25.2	25.0–27.3	26.2 ± 0.7
PFL	%SL	27.4	24.2–29.3	27.4 ± 1.3
CPL	%SL	19.4	17.8–22.6	20.2 ± 1.1
CPD	%SL	9.0	8.5–10.4	9.6 ± 0.5
CFL	%SL	23.9	22.2–25.2	24.4 ± 0.8
HL	%SL	30.5	31.0–34.6	32.9 ± 0.9
SnL	%HL	26.6	23.7–29.7	26.4 ± 1.3
SnW	%HL	26.1	24.1–27.8	25.8 ± 1.2
HW	%HL	42.9	37.2–44.2	40.6 ± 1.7
IOW	%HL	21.7	19.3–21.7	20.7 ± 0.8
POW	%HL	26.1	22.1–27.3	24.1 ± 1.4
LaW	%HL	22.3	19.8–24.4	22.0 ± 1.3
POD	%HL	13.6	11.7–18.0	14.4 ± 1.8
EyL	%HL	35.9	32.7–40.2	36.5 ± 2.2
EyD	%HL	34.2	28.5–35.0	32.1 ± 1.8
ChD	%HL	14.1	13.1–16.3	15.0 ± 1.1
LJL	%HL	42.1	39.0–45.6	41.0 ± 1.6
LJW	%HL	12.5	11.7–18.0	14.0 ± 1.5
EyD/EyL		1.0	0.8–0.9	0.9 ± 0.0
LJL/LJW		3.4	2.2–3.6	3.0 ± 0.3

**Habitus.** See Fig. 6. Body slender. Dorsal head profile straight to slightly curved. Premaxillary pedicel slightly prominent. Mouth oblique. Lips not thickened. Medial part of premaxilla slightly expanded to expanded. Caudal part of maxilla not bullate. Vertical through caudal tip of maxilla running through iris, just rostral to pupil. Lateral snout outline isognathous and obtuse, in large specimens sometimes slightly prognathous. Jaws equal anteriorly or lower jaw slightly protruding. Mental prominence slightly pronounced. Retro-articular processes of right and left mandible touching each other, interrupting ventral body outline. Eye approximately circular (occasionally slightly elongated) and medium to large in size. Distinct aphakic aperture present in pupil. Cephalic lateral line pores not enlarged.

**Scales.** Cheek, gill cover, and rostral part of the dorsal head surface with cycloid scales. Nape and rostral part of dorsum with a mixture of cycloid and weakly ctenoid scales. Chest mainly with ctenoid scales, occasionally weakly ctenoid or cycloid. Scales on remaining part of body mainly ctenoid. Scales on chest smaller than those on ventral and ventro-lateral part of body; size transition gradual. Small elongated scales on basal quarter to one third of caudal fin. Four to 5.5 (mode 5) scales between upper lateral line and dorsal-fin origin, four to seven (mode 6) between between pectoral- and pelvic-fin bases.

**Fins.** Pelvic fins just reaching or slightly surpassing rostral-most point of anal-fin origin. Pelvic fins with first soft rays slightly produced, occasionally filamentous. Caudal tip of anal fin not reaching caudal-fin origin. Caudal-fin outline truncate to slightly emarginate.

**Gill apparatus.** Description of gill apparatus based on lateral gill rakers and lateral hemibranch of first gill arch. Number of gill rakers on lower part of first gill arch 10–12 (one specimen with 13). Lower two to three rakers reduced (= very short), next one to two short, followed by two to seven slender and longer ones. Remaining rakers hooked, bifid, or trifid. Generally rakers closely set, viz. touching each other over major part of length. Number of gill filaments 87 to 93.

**Viscera.** Ratio between intestine length and SL: 1.0–1.2 (n = 5).

**Figure 7. F7:**
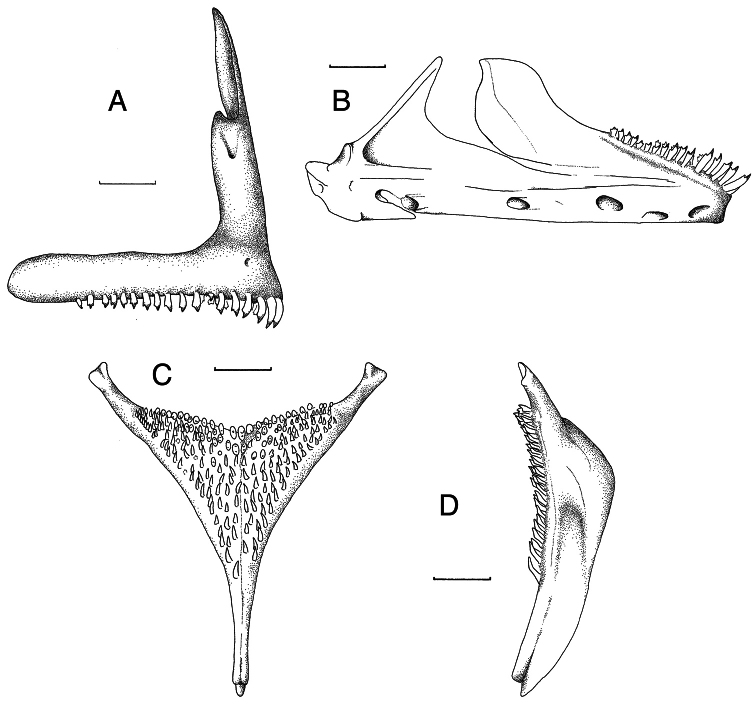
Skeletal elements of *Haplochromis goldschmidti* sp. n. **A** Right premaxilla, lateral view (RMNH.PISC.83695) **B** Right lower jaw, lateral view (RMNH.PISC.83695) **C** Lower pharyngeal element, dorsal view (RMNH.PISC.83701) **D** Lower pharyngeal element, lateral view (RMNH.PISC.83701). Scale bars equal 1 mm. Drawn by I. Westbroek.

**Oral jaws.** (Fig. 7 A, B) Premaxillary ascending arm equal to or longer than dentigerous arm (asc./dent. arm ratio 1.0 to 1.1). Angle between arms 77° to 81°. Symphyseal articulation facet not present, lower jaw slightly more elongated than generalized type (length/height ratio 2.3 to 2.8). Upper half of dentary with distinct outwardly directed flare. Mental prominence slightly pronounced.

**Oral teeth shape.** (Fig. 7 A, B) Teeth of outer row in both jaws mainly bicuspid, with some unicuspid or tricuspid teeth interspersed. Major cusp of bicuspids isoscelene to subequilateral, protracted and acutely pointed. Flange occasionally present on major cusp. Minor cusp short compared to major cusp. Cusp gap rather narrow. In labial view neck moderately slender to normal, crown moderately expanded. In lateral view, crown compressed. Outer-row teeth in both premaxilla and lower jaw recurved. Inner rows in both jaws with mainly tricuspid or weakly tricuspid teeth.

**Oral teeth size.** Outer-row teeth relatively slender, gradually decreasing in size from rostral to caudal.

**Dental arcade and tooth band.** Rostrally dental arcade rounded. Outer row occupying ^3^/_4_ to ^4^/_5_ of premaxillary dentigerous arm. One to two inner rows in both jaws.

**Teeth counts and setting.** Outer row of upper jaw (l+r premaxilla) with 33–47 teeth. In both jaws outer-row teeth regularly set. Teeth set wider rostrally than laterally.

**Tooth implantation.** Outer-row teeth of premaxilla rostrally erect. Inner-row teeth recumbent. Outer-row teeth of lower jaw slightly procumbent, inner-row teeth erect.

**Lower pharyngeal element.** (Fig. 7 C, D) Lower pharyngeal element relatively small and slender (length/width ratio 1.2–1.3). Dentigerous area slightly broader than long (length/width ratio 0.7–0.9). Suture straight.

**Pharyngeal teeth** **counts.** Caudal-most transverse row with 25–34 teeth, medial longitudinal rows with nine to 10 teeth.

**Pharyngeal teeth shape.** Teeth in caudal-most transverse row hooked, major cusp only slightly incurved, blunt to slightly acute. Other teeth bevelled or pronounced. All teeth relatively fine and slender, medial teeth not coarser than other teeth.

**Vertebrae.** Total number of vertebrae in 19 specimens: 30 (2), 31 (16) or 32 (1), comprising 13–14 abdominal and 16–18 caudal vertebrae.

**Figure 8. F8:**
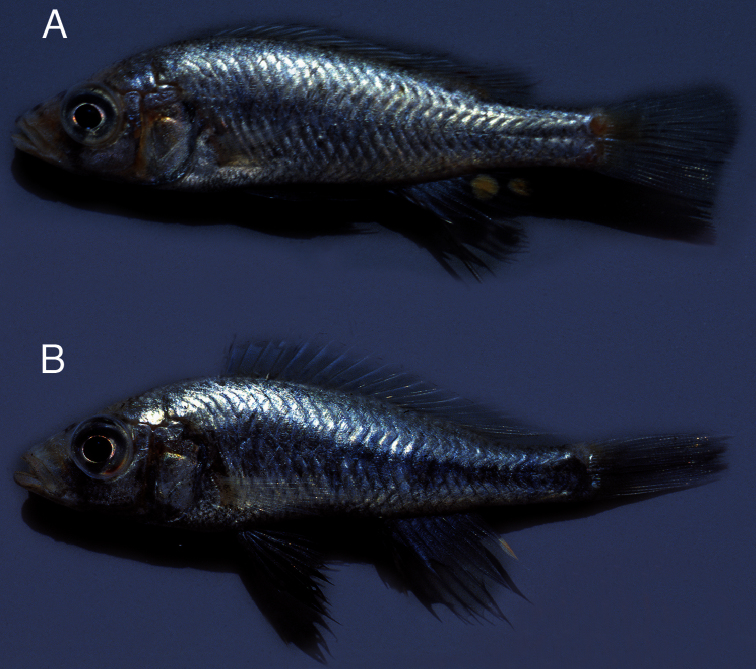
Live colours of *Haplochromis goldschmidti* sp. n. **A** sexually active ♂, 53.6 mm SL (paratype, RMNH.PISC.80480) **B** sexually active ♂, 57.5 mm SL (paratype, RMNH.PISC.83575).

**Live colouration males.** (Fig. 8 A, B) Sexually active males with grey-white snout, cheek and gill cover. Lips grey-white, generally with distinct black pigment spots. Eye with grey outer ring and silver inner ring. Dorsal head surface and dorsum silvery-grey. Chest, ventral side, flank and ventral part of caudal peduncle silvery-grey with dusky flush. Flush most distinct on flank and caudal peduncle and occasionally absent on ventral side, sometimes extending over suboperculum, interoperculum, branchiostegal membrane and lower jaw.

Pelvic fins black. Anal fin rostrally hyaline-grey with bluish sheen, remaining part hyaline. One to two pale-yellow to yellow egg dummies surrounded by hyaline ring on caudal part of anal fin. Caudal fin hyaline with bluish sheen; dusky flush on caudal peduncle may extend over rostral part of caudal fin. Dorsal fin hyaline with bluish sheen and faint dusky lappets.

Dark grey to blackish markings: Faint nostril-, interorbital- and supraorbital stripes sometimes present. Lachrymal stripe occasionally slightly longer than in *Haplochromis argens*, but often less distinct. Preopercular vertical stripe generally not clear. Opercular blotch present.

**Preserved colouration of**

##### males.

**Figure 9.**
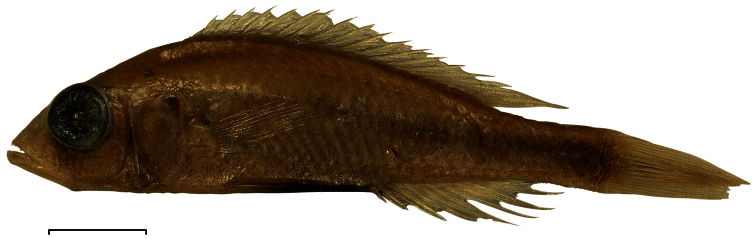
Preserved colours of *Haplochromis goldschmidti* sp. n. ♂ 60.3 mm SL (holotype, RMNH.PISC.83573). Scale bar equals 10 mm.

(Fig. 9) Body light brown. Chest, ventral side, flank and ventral part of caudal peduncle dusky to dark brown. Dark brown colour on caudal peduncle sometimes giving impression of broad mid-lateral band. Fins transparent to light grey-brown except for pelvics, which are black in adult males. Same markings present as in live specimens.

##### Distribution.

*Haplochromis goldschmidti* is only known from the southern part of the Emin Pasha Gulf of Lake Victoria (Fig. 1).

##### Habitat.

*Haplochromis goldschmidti* was caught over mud bottoms at depths of 4–10 m.

##### Food.

Stomach and intestines of five examined specimens caught by day contained mainly zooplankton (mainly copepods, but also some cladocerans) and some insects (*Chaoborus* larvae and pupae).

##### Breeding.

Based on the egg dummies on the anal fin of males, *Haplochromis goldschmidti* is probably a female mouth brooder.

##### Etymology.

This species is named in honour of Dr Tijs Goldschmidt in appreciation for his work on haplochromine cichlids of Lake Victoria. As a member of the *Haplochromis* Ecology Survey Team, Tijs Goldschmidt worked in Tanzania (1981–1986) on the ecology and evolution of zooplanktivorous and detritivorous cichlids. *Haplochromis goldschmidti* is one of the species on which he based his theory on the possible role of egg-dummy divergence in speciation of haplochromines (Goldschmidt and de Visser 1990). With “Darwin’s Dreampond” (Goldschmidt 1996) - originally published as “Darwins Hofvijver” (Godschmidt 1994) and translated in eight languages - Tijs Goldschmidt started his career as a writer and brought the human-induced extinction of the Lake Victoria cichlids to the attention of a worldwide public. The specific epithet, *goldschmidti*,is a Latinized version (genitive case) of the surname.

##### Comparisons.

Sexually active males of *Haplochromis goldschmidti* and *Haplochromis argens* are very similar, but distinguishable by live colouration. *Haplochromis goldschmidti* has a dusky flush on its flank and *Haplochromis argens* a yellow to greenish sheen. In contrast to *Haplochromis argens*, red is generally absent on the fins of *Haplochromis goldschmidti*, only occasionally very faint traces of red are present on the caudal and dorsal fins. *Haplochromis goldschmidti* has plain yellow egg spots, while the egg spots of *Haplochromis argens* are orange yellow (Goldschmidt and de Visser 1990). Generally, *Haplochromis goldschmidti* has more pigment spots on its lips than *Haplochromis argens*.The body depth in *Haplochromis goldschmidti* is less deep than in *Haplochromis argens*. The snout width, lachrymal width and lower jaw width are generally larger in *Haplochromis goldschmidti* (Tables 1, 3, 4).

**Table 4.** Adjusted means (rounded to the nearest 0.1 mm), their differences (in %) and significance levels of the ANCOVAs of linear measurements. Both populations of *Haplochromis argens* are compared to *Haplochromis goldschmidti*. The mean values represent antilogged adjusted means calculated from the ANCOVA analyses (sample mean adjusted for a common mean standard length and a common regression line for the three groups). Adjusted means and differences are not applicable (NA) when the slopes of the relationships differ (i.e., in case of a significant interaction between location and SL). Estimated differences were calculated from adjusted means. Parameter estimates were derived from the GLM (analysis of covariance) procedure. Significance levels (*P*) of the effect of species (Spec.), location (Loc.) and the interaction between SL and location (Loc. * SL) are given when below 0.05.

	*Haplochromis argens*	*Haplochromis argens*	*Haplochromis goldsch*	*Haplochromis argens*	*Haplochromis argens*	Spec.	Loc.	Loc. * SL
	**Mwanza**	**Emin P**	**Emin P**	**Mwanza**	**Emin P**			
	**Mean**	**Mean**	**Mean**	**% diff.**	**% diff.**	**P**	**P**	**P**
BD	NA	NA	NA	NA	NA	< 0.001	0.041	0.031
PFL	17.6	16.9	16.9	4.1	0.0	ns	< 0.001	ns
CPL	12.9	12.9	12.4	3.3	3.3	0.012	ns	ns
CPD	6.3	6.0	6.0	5.5	0.0	ns	< 0.001	ns
CFL	15.0	14.6	15.1	-0.6	-3.2	0.007	0.016	ns
HL	20.4	20.4	20.4	0.0	0.0	ns	ns	ns
SnL	5.4	5.4	5.4	0.0	0.0	ns	ns	ns
SnW	5.1	4.8	5.3	-2.7	-9.0	< 0.001	< 0.001	ns
HW	NA	NA	NA	NA	NA	ns	< 0.001	< 0.001
IOW	4.3	4.2	4.2	2.7	0.0	ns	0.011	ns
POW	4.7	4.7	4.9	-3.5	-3.5	0.004	ns	ns
LaW	4.2	4.0	4.5	-7.3	-10.4	< 0.001	0.046	ns
POD	3.1	3.1	2.9	5.4	5.4	0.030	ns	ns
EyL	NA	NA	NA	NA	NA	ns	< 0.001	< 0.001
EyD	6.5	6.5	6.5	0.0	0.0	ns	ns	ns
ChD	3.5	3.1	3.1	11.7	0.0	ns	< 0.001	ns
LJL	NA	NA	NA	NA	NA	ns	0.039	0.040
LJW	2.8	2.6	2.8	-2.1	-9.1	0.001	0.003	ns

Sexually active males of *Haplochromis goldschmidti* are distinguished from *Haplochromis tanaos* and *Haplochromis thereuterion*, two other slender zooplanktivores, by colouration. A distinct mid- and dorsal-lateral band is absent in males of *Haplochromis goldschmidti*, whereas they are present in *Haplochromis tanaos* and *Haplochromis thereuterion* (sometimes difficult to distinguish in the latter). A nape band is lacking in *Haplochromis goldschmidti*, but present in *Haplochromis tanaos*.

### Comparison of species and populations

The MANCOVA of the linear measurements of males of *Haplochromis goldschmidti* and both populations of *Haplochromis argens* shows that SL is a significant covariate (*P* < 0.001), explaining the largest part of the variance (*F* = 81.406). The two species differ significantly (species effect, *P* < 0.001), and the same holds for the populations from the Mwanza Gulf and the Emin Pasha Gulf (location effect, *P* = 0.007), with the effect of species explaining more of the variance (*F* = 9.262) than the effect of location (*F* = 2.494). There is also a significant interaction between SL and location, however it does not explain much of the variance (*F* = 2.545).

From the ANCOVAs of the individual measurements, the adjusted means and their differences were calculated (Table 4).

**Figure 10.**
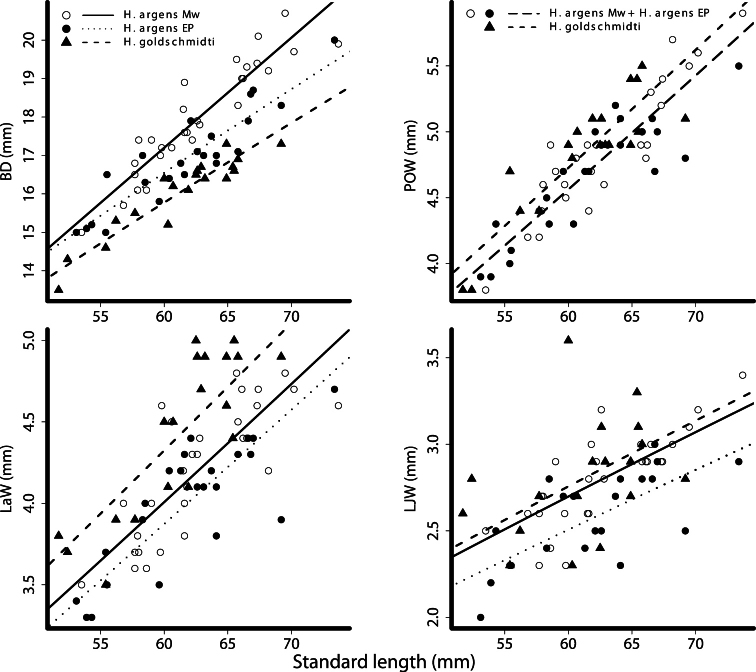
Plots of taxonomic measurements as a function of standard length. Body depth (BD), preorbital width (POW), lower jaw width (LJW) and lachrymal width (LaW). Open circles - *Haplochromis argens* from the Mwanza Gulf (Mw); closed circles - *Haplochromis argens* from the Emin Pasha Gulf (EP); triangles - *Haplochromis goldschmidti*. Power curves determined from GLM parameter estimates, solid line - *Haplochromis argens* from the Mwanza Gulf; dotted line - *Haplochromis argens* from the Emin Pasha Gulf; dashed line - *Haplochromis goldschmidti*, long dashed line (POW) - *Haplochromis argens* from both locations (MW + EP).

There is a significant species effect in eight (BD, CPL, CFL, SnW, POW, LaW, POD and LJW) of the 18 measurements, and in three of these measurements (CPL, POW, POD) this is the only significant effect. The other measurements (BD, CFL, SnW, LaW and LJW) also differ significantly between the two populations of *Haplochromis argens* (= effect of location). In addition, for BD, a significant interaction (*P* = 0.031) between the effects of location and SL indicate an increasing relative difference in BD (with increasing standard length) between the estimations for the populations from the different locations (Table 4, Fig. 10).

In the measurements, except for BD, in which there is a significant effect of species and location, the population of *Haplochromis argens* from the Emin Pasha Gulf differs more from *Haplochromis goldschmidti* than does the population of *Haplochromis argens* from the Mwanza Gulf. Especially in width measurements from the oral and suspensorial compartment, SnW, LaW and LJW, this is apparent (Fig. 10). In BD, *Haplochromis argens* from the Mwanza Gulf differs more from *Haplochromis goldschmidti* than does *Haplochromis argens* from the Emin Pasha Gulf (Fig. 10).

The effect of location is significant in 12 of the 18 measurements, and for PFL, CPD, IOW and ChD, this is the only significant effect; these measurements are significantly larger in *Haplochromis argens* from the Mwanza Gulf than in *Haplochromis argens* from the Emin Pasha Gulf and *Haplochromis goldschmidti*. In four measurements in which there is a significant effect of location, the two-way interaction is significant as well. For BD, HW and LJL, the relative difference between *Haplochromis argens* from the Mwanza Gulf and both *Haplochromis argens* and *Haplochromis goldschmidti* from the Emin Pasha Gulf increases with SL. For EyL, however, the relative difference between *Haplochromis argens* from the Mwanza Gulf and both *Haplochromis argens* and *Haplochromis goldschmidti* from the Emin Pasha Gulf decreases with SL.

## Discussion

### Females of *Haplochromis goldschmidti*

In this paper, females of *Haplochromis goldschmidti* have not been described since it was difficult to distinguish the females from females of *Haplochromis argens* in the field. Samples of females, which presumably contained both *Haplochromis argens* and *Haplochromis goldschmidti*, were collected during ecological surveys in the Emin Pasha Gulf, but unfortunately these samples were mislaid or may have been lost during the many moves of the material since its collection.

### Specific status of *Haplochromis goldschmidti*

Since the field research of Greenwood (many papers bundled in 1981), male colouration has become a major character in species distinction of Lake Victoria cichlids. Indeed, experimental research has shown that male colouration plays a major role in female mate choice of Lake Victoria haplochromines (Seehausen and van Alphen 1998). However, Seehausen et al. (1998, 1999) also found some rock-dwelling species with a distinct polymorphism in breeding colours of sympatrically (and allopatrically) occurring males. These males did not differ in morphological characters, and mating experiments in tanks confirmed these colour polymorphisms (Seehausen and Bouton 1996; Seehausen et al. 1999). Nevertheless, we consider it unlikely that *Haplochromis argens* and *Haplochromis goldschmidti* represent an example of a species with male colour polymorphism, as apart from male body colouration there are at least four other presumably independent characters that differ in the two species (viz.: colour, position and number of egg spots (Goldschmidt and de Visser 1990); width measures of the head; body depth; differences in tooth shape). Based on the genotypic cluster species concept of Mallet (1995) it has been suggested that groups of haplochromine cichlids that differ in male nuptial colouration and one or more other characters that are likely to be genetically independent (e.g. dentition, body shape etc.) will be considered as different species (Seehausen et al. 1998). As will be clear from the comparison above, *Haplochromis argens* and *Haplochromis goldschmidti* fulfil these criteria.

### Generic classification

Greenwood (1979, 1980) revised the Lake Victoria haplochromines and split them into more than 20 genera and subgenera. During the past decades there have been extensive debates on the validity of the genera defined by Greenwood (Hoogerhoud 1984; Meyer et al. 1990; Lippitsch 1993; Snoeks 1994; Seehausen 1996; van Oijen 1996; Seehausen et al. 1998). Because of the disagreements, and as a considerable number of the haplochromine species from Lake Victoria cannot be assigned to the “new” genera, we prefer to keep newly described species in the genus *Haplochromis* and add the generic names from Greenwood’s revision of the Lake Victoria haplochromines (Greenwood 1979, 1980) between brackets in cases where the assignment is unequivocal.

In some studies *Haplochromis argens* has been assigned to *Yssichromis* (e.g. van Staaden et al. 1995; Chapman et al. 1995; Huber et al. 1997; Rosenberger and Chapman 2000; Melnychuk and Chapman 2002). However, even when adopting Greenwood’s revision, for the following reasons we doubt whether this allocation to *Yssichromis* is correct. According to Greenwood (1980: 24) “*Yssichromis* is an isolated lineage, defined by its autapomorphic features (shallow elongate body, and posteriorly edentulous premaxilla)”. Though *Haplochromis argens* has a shallow, elongate body, it generally lacks a posteriorly edentulous premaxilla. Moreover, compared to the zooplanktivorous species assigned to *Yssichromis*, *Haplochromis argens* has a more piscivorous facies with some features that resemble those of *Prognathochromis*, e.g. a slightly prognathous lower jaw, a slightly pronounced mental prominence, the medial part of the premaxilla slightly expanded. Further, its oral teeth are longer and have a more protracted and acute major cusp than the teeth of the zooplanktivores that were assigned to *Yssichromis*. Greenwood (1980: 23) wrote concerning *Yssichromis*: “Superficially, members of this genus resemble the *Prognathochromis* lineage, especially members of the subgenus *Tridontochromis*. However, *Yssichromis* species retain several generalized features in the syncranium, and the lower jaw length is shorter, although some overlap does occur.” The lower jaw of *Haplochromis argens* is indeed too short for the species to be recognized as *Prognathochromis* (viz. 37.6–44.3% of HL, mean 40.5% versus 41–62%, modal range 45–53%), and judging from the radiographs, the neurocranium is of the generalized type. Based on the information above, the position of *Haplochromis argens* seems to be between *Yssichromis* and *Prognathochromis*.

*Haplochromis goldschmidti* has the two autapomorphic characters of *Yssichromis* and can thus be referred to as *Haplochromis (Yssichromis) goldschmidti*. However, in some characters it seems to bridge the gap between *Haplochromis argens* and the species of the *Yssichromis* group, as its teeth are longer than those of the latter, but shorter than those of *Haplochromis argens*, and it has the medial part of the premaxilla slightly expanded to expanded. Both *Haplochromis argens* and *Haplochromis goldschmidti* lack the typical arrangement of the inner rows in the lower jaw described for *Haplochromis (Yssichromis) laparogramma*, *Haplochromis (Yssichromis) pyrrhocephalus* and *Haplochromis (Yssichromis) heusinkveldi* by Witte and Witte-Maas (1987). These species have a single inner row except for the rostro-lateral corner, where two inner rows are present.

Considering the resemblance between *Haplochromis argens* and *Haplochromis cassius*, the latter assigned to the genus *Psammochromis* by Greenwood (1980), we investigated whether *Haplochromis argens* could be assigned to this genus. The main apomorpic character for *Psammochromis*, a dentary with each ramus inflated anteriorly and antero-laterally (Greenwood 1980), has not been found in *Haplochromis argens* (and also not in *Haplochromis goldschmidti*). Other diagnostic characters for the genus, like thickened lips and a maximum adult size of 100–123 mm SL, have been found in neither *Haplochromis argens* nor in *Haplochromis goldschmidti*. So there are no arguments for assigning the species to *Psammochromis*.

### Comparison of *Haplochromis goldschmidti* with the populations of *Haplochromis argens*

In some morphological characters (e.g. body depth), *Haplochromis goldschmidti* is more similar to the sympatric population of *Haplochromis argens* than to the allopatric population of the latter in the Mwanza Gulf, whereas for other characters (snout width, lower jaw width) the opposite is true. It has been found in earlier studies that local adaptations related to environmental conditions may cause similar morphological shifts in several species of Lake Victoria haplochromines (Bouton et al. 1999, 2002). A recent study by van Rijssel and Witte (2012) revealed a decrease in body depth of two resurgent zooplanktivorous species in the Mwanza Gulf in the 1990s, which they associated with increased predation pressure by Nile perch in that period. Local environmental conditions may result in morphological similarity between sympatric populations of different species. On the other hand, competition for food might provide a tentative explanation for the greater difference in width of the snout area between *Haplochromis goldschmidti* and the sympatric population of *Haplochromis argens* in the Emin Pasha Gulf. However, to confirm this, information would be needed about their feeding habits in the areas where the two species coexist and where *Haplochromis argens* occurs alone.

### Endangered species

Eight zooplanktivorous haplochromine species that were common on our research transect in the Mwanza Gulf (Goldschmidt et al. 1990) had virtually disappeared by 1987 (Witte et al. 1992b). However, since the early 1990s a gradual recovery has been observed for three of these species: *Haplochromis pyrrhocephalus*, *Haplochromis laparogramma* and *Haplochromis tanaos* (Seehausen et al. 1997b, Witte et al. 2000). Since 2005, these species are even more common than they used to be in the 1970s (Witte et al. 2007a, 2012a, Kishe-Machumu 2012). In contrast, the other five species, including *Haplochromis argens*, remain rare or are absent. In 2002, *Haplochromis argens* was observed for the first time since its disappearance in 1987, and since then it has been caught only occasionally in the Mwanza Gulf (Table 2) and in the Speke Gulf (Mizoiri et al. 2008) in spite of intensive sampling programmes. The catch per unit effort on the research transect has declined more than 50 times, and it is likely that the species is in danger of extinction. We are not sure of the conservation status of *Haplochromis goldschmidti*, as we did not sample the Emin Pasha Gulf, the only area from which the species is known, after 1986. This restricted distribution makes the species vulnerable in any case.

In the successfully recovering zooplanktivorous haplochromine species, morphological changes were observed, which may have been caused by adaptive responses to the environmental changes, both through natural selection and phenotypic plasticity (Wanink and Witte 2000, Witte et al. 2008, Chapman et al. 2008, van der Meer et al. 2012; van Rijssel and Witte 2012). In some cases (introgression through) hybridization may have also taken place among zooplanktivores (Mzighani et al. 2010). Van Rijssel and Witte (2012) found that several successfully resurgent haplochromine species had a smaller head-surface area/caudal-peduncle area than in the past, which aids in predator escape because it facilitates burst swimming (Langerhans 2010). In the not or poorly recovering zooplanktivorous species *Haplochromis heusinkveldi*, and *Haplochromis piceatus* Greenwood & Gee, 1969, the head-surface area/caudal-peduncle area changed in the opposite direction (van Rijssel and Witte 2012). A preliminary study of the few individuals that were collected in the Mwanza Gulf in the 2000s suggests that this also was the case in *Haplochromis argens*. These changes in the ratio between head surface and caudal peduncle will be further studied when more material becomes available in the future. Obviously, the recovering zooplanktivores are of great interest for future research, and the species descriptions in this paper contribute to the taxonomic baseline for these studies.

## Supplementary Material

XML Treatment for Haplochromis argens

XML Treatment for Haplochromis goldschmidti

## References

[B1] Anker GCh Dullemeijer P (1996) Transformation morphology on structures in the head of cichlid fishes. In: Datta Munsghi JS Dutta HM (Eds). Fish Morphology – Horizon of New Research. Oxford & IBH Publishing, New Delhi: 1-20.

[B2] Balshine-Earn S (1995) The costs of parental care in Galilee St Peter’s fish, *Sarotherodon galilaeus*. Animal Behaviour 50: 1-7. doi: 10.1006/anbe.1995.0214

[B3] Barel CDN (1983) Towards a constructional morphology of cichlid fishes (Teleostei, Perciformes). Netherlands Journal of Zoology 33: 357-424. doi: 10.1163/002829683X00183

[B4] Barel CDN (1985) A matter of space, constructional morphology of cichlid fishes. PhD Thesis, Leiden, the Netherlands: Leiden University.

[B5] Barel CDN (1993) Concepts of an architectonic approach to transformation morphology. Acta Biotheoretica 41: 345-381. doi: 10.1007/BF00709371

[B6] Barel CDN Ligtvoet W Goldschmidt T Witte F Goudswaard PC (1991) The haplochromine cichlids in Lake Victoria: an assessment of biological and fisheries interests. In: Keenleyside MHA (Ed). Cichlid Fishes, Behaviour, Ecology and Evolution. Chapman & Hall, London: 258-279.

[B7] Barel CDN van Oijen MJP Witte F Witte-Maas ELM (1977) An introduction to the taxonomy and morphology of the haplochromine Cichlidae from Lake Victoria. Netherlands Journal of Zoology 27: 333-389.

[B8] Barel CDN Witte F van Oijen MJP (1976) The shape of the skeletal elements in the head of a generalized Haplochromis species: H. elegans Trewavas 1933 (Pisces, Cichlidae). Netherlands Journal of Zoology 26: 163-265. doi: 10.1163/002829676X00019

[B9] Bouton N de Visser J Barel CDN (2002) Correlating head shape with ecological variables in rock-dwelling haplochromines (Teleostei: Cichlidae) from Lake Victoria. Biological Journal of the Linnean Society 76: 39-48. doi: 10.1111/j.1095-8312.2002.tb01712.x

[B10] Bouton N Witte F van Alphen JJM Schenk A Seehausen O (1999) Local adaptations in populations of rock-dwelling haplochromines (Pisces: Cichlidae) from southern Lake Victoria. Proceedings of the Royal Society of London B 266: 355-360. doi: 10.1098/rspb.1999.0645

[B11] Carleton KL Spady TC Streelman JT Kidd MR McFarland WN Loew ER (2008) Visual sensitivities tuned by heterochronic shifts in opsin gene expression. BioMed Central Biology 6: 22. doi: 10.1186/1741-7007-6-22 PMC2430543 18500997

[B12] Chapman LJ Chapman CA Kaufman L Witte F Balirwa J (2008) Biodiversity conservation in African inland waters: lessons of the Lake Victoria region. Verhandlungen Internationale Vereinigung für theoretische und angewandte Limnologie 30: 16-34.

[B13] Chapman LJ Kaufman LS Chapman CA McKenzie FE (1995) Hypoxia tolerance in twelve species of East African cichlids: potential for low oxygen refugia in Lake Victoria. Conservation Biology 9: 1274-1287. doi: 10.1046/j.1523-1739.1995.9051262.x-i1 34261244

[B14] Collin SP Hart NS Wallace KM Shand J Potter IC (2004) Vision in the southern hemisphere lamprey *Mordacia mordax*: Spatial distribution, spectral absorption characteristics, and optical sensitivity of a single class of retinal photoreceptor. Visual Neuroscience 21: 765–773. doi: 10.1017/S0952523804215103 15683562

[B15] de Visser J (2000) Tongue in cheek. A study in biological engineering of fish. PhD Thesis, Leiden, the Netherlands: Leiden University.

[B16] de Visser J Barel CDN (1996) Architectonic constraints on the hyoid’s optimal starting position for suction feeding of fish. Journal of Morphology 228: 1-18. doi: 10.1002/(SICI)1097-4687(199604)228:1<1::AID-JMOR1>3.0.CO;2-B 29852570

[B17] Goldschmidt T (1989a) An ecological and morphological field study on the haplochromine cichlid fishes (Pisces, Cichlidae) of Lake Victoria. PhD Thesis, Leiden, the Netherlands: Leiden University.

[B18] Goldschmidt T (1989b) Reproductive strategies, subtrophic niche differentiation and the role of competition for food in haplochromine cichlids (Pisces) from Lake Victoria, Tanzania. Annalenvan het Koninklijk Museum voor Midden-Afrika, Zoologische Wetenschappen 257: 119-132.

[B19] Goldschmidt T (1991) Egg mimics in haplochromine cichlids (Pisces, Perciformes) from Lake Victoria. Ethology 88: 177-190. doi: 10.1111/j.1439-0310.1991.tb00273.x

[B20] Goldschmidt T (1994) Darwins Hofvijver. Uitgeverij Prometheus, Amsterdam, 286 pp.

[B21] Goldschmidt T (1996) Darwin’s Dreampond. MIT Press, Cambridge, Massachusetts, 277 pp.

[B22] Goldschmidt T de Visser J (1990) On the possible role of egg mimics in speciation. Acta Biotheoretica 38: 125-134. doi: 10.1007/BF00047549

[B23] Goldschmidt T Witte F (1990) Reproductive strategies of zooplanktivorous haplochromine species (Pisces, Cichlidae) from Lake Victoria before the Nile perch boom. Oikos 58: 356-368. doi: 10.2307/3545227

[B24] Goldschmidt T Witte F (1992) Explosive speciation and adaptive radiation of haplochromine cichlids from Lake Victoria: an illustration of the scientific value of a lost species flock. Mitteilungen Internationale Vereinigung für theoretische und angewandte Limnologie 23: 101–107.

[B25] Goldschmidt T Witte F de Visser J (1990) Ecological segregation of zooplanktivorous haplochromine species (Pisces, Cichlidae) from Lake Victoria. Oikos 58: 343-355. doi: 10.2307/3545226

[B26] Goldschmidt T Witte F Wanink JH (1993) Cascading effects of the introduced Nile perch on the detritivorous/phytoplanktivorous species in the sublittoral areas of Lake Victoria. Conservation Biology 7: 686-700. doi: 10.1046/j.1523-1739.1993.07030686.x

[B27] Gottfried MD (1986) Developmental transition in feeding morphology of the Midas cichlid. Copeia 1986: 1028-1030. doi: 10.2307/1445308

[B28] Goudswaard K (PC) Witte F Katunzi EFB (2008) The invasion of an introduced predator, Nile perch (*Lates niloticus*, L.) in Lake Victoria (East Africa): chronology and causes. Environmental Biology of Fishes 81: 127-139. doi: 10.1007/s10641-006-9180-7

[B29] Greenwood PH (1979) Towards a phyletic classification of the ‘genus’ *Haplochromis* and related taxa. Part I. Bulletin British Museum of natural History (Zoology) 35: 265-322.

[B30] Greenwood PH (1980) Towards a phyletic classification of the ‘genus’ *Haplochromis* and related taxa. Part II. Bulletin British Museum of natural History (Zoology) 39: 1-101.

[B31] Greenwood PH (1981) The Haplochromine Fishes of the East African Lakes. Kraus International Publications, München, 839 pp.

[B32] Greenwood PH Barel CDN (1978) A revision of the Lake Victoria *Haplochromis* species (Pisces, Cichlidae), Part VIII. Bulletin British Museum of natural History (Zoology), 33: 141–192.

[B33] Hecky RE (1993) The eutrophication of Lake Victoria. Verhandlungen Internationale Vereinigung für theoretische und angewandte Limnologie 25: 39-48.

[B34] Hecky RE Bugenyi FWB Ochumba P Talling JF Mugidde R Gophen M Kaufman L (1994) Deoxygenation of the deep water of Lake Victoria, East Africa. Limnology and Oceanography 39: 1476-1481. doi: 10.4319/lo.1994.39.6.1476

[B35] Hecky RE Mugidde R Ramlal PS Talbot MR Kling GW (2010) Multiple stressors cause rapid ecosystem change in Victoria. Freshwater Biology 55 (1): 19-42. doi: 10.1111/j.1365-2427.2009.02374.x

[B36] Hilder ML Pankhurst NW (2003) Evidence that temperature change cues reproductive development in the spiny damselfish, *Acanthochromis polyacanthus*. Environmental Biology of Fishes 66: 187-196. doi: 10.1023/A:1023601729203

[B37] Hoogerhoud RJC (1984) A taxonomic reconsideration of the haplochromine genera *Gaurochromis* Greenwood, 1980 and *Labrochromis* Regan, 1920 (Pisces, Cichlidae). Netherlands Journal of Zoology 34: 539-565. doi: 10.1163/002829684X00281

[B38] Hoogerhoud RJC Witte F (1981) Revision of species from the ‘Haplochromis’ empodisma group. Netherlands Journal of Zoology 31: 232-273. doi: 10.1163/002829680X00258

[B39] Huber R van Staaden MJ Kaufman LS Liem KF (1997) Microhabitat use, trophic patterns, and the evolution of brain structure in African cichlids. Brain, Behaviour and Evolution 50: 167-182. doi: 10.1159/000113330 9288416

[B40] Kaufman L (1992) Catastrophic change in species-rich freshwater ecosystems. BioScience 42: 846–858. doi: 10.2307/1312084

[B41] Kaufman L Seehausen O (1995) Cichliden von der ugandischen und der kenianischen Victoriaseeküste. In: Stawikowski R (Ed). Cichliden, Festschrift zum 25jährigen Jubiläum der DCG. Deutsche Cichliden-Gesellschaft, Frankfurt am Main: 147-151.

[B42] Katunzi EFB Zoutendijk J Goldtschmidt T Wanink JH Witte F (2003) Lost zooplanktivorous cichlid from Lake Victoria reappears with a new trade. Ecology of Freshwater Fish 12: 237-240. doi: 10.1046/j.1600-0633.2003.00023.x

[B43] Kishe-Machumu MA (2012) Inter-guild differences and possible causes of the recovery of cichlid species in Lake Victoria, Tanzania. PhD Thesis, Leiden, the Netherlands: Leiden University.

[B44] Kishe-Machumu M Witte F Wanink JH (2008) Dietary shift in benthivorous cichlids after the ecological changes in Lake Victoria. Animal Biology 58: 401-417. doi: 10.1163/157075608X383700

[B45] Langerhans RB (2010) Predicting evolution with generalized models of divergent selection: a case study with poeciliid fish. Integrative and Comparative Biology 50: 1167-1184. doi: 10.1093/icb/icq117 21558265

[B46] Lippitsch E (1993) A phyletic study on lacustrine haplochromine fishes (Perciformes, Cichlidae) of East Africa, based on scale and squamation characters. Journal of Fish Biology 42: 903–946. doi: 10.1111/j.1095-8649.1993.tb00399.x

[B47] Mallet J (1995) A species definition for the modern synthesis. Trends in Ecology & Evolution 10: 294-299. doi: 10.1016/0169-5347(95)90031-4 21237047

[B48] Melnychuk MC Chapman LJ (2002) Hypoxia tolerance of two haplochromine cichlids: swamp leakage and potential for interlacustrine dispersal. Environmental Biology of Fishes 65: 99-110. doi: 10.1023/A:1019602403098

[B49] Meyer A Kocher TD Basasibwaki P Wilson AC (1990) Monophyletic origin of Lake Victoria cichlid fishes suggested by mitochondrial DNA sequences. Nature 347: 550-553. doi: 10.1038/347550a0 2215680

[B50] Mizoiri S Aibara M Okada N (2008) Live Cichlids in the Southern Lake Victoria - Ongoing Speciation. (in Japanese) Report of Grant-in-Aid for Scientific Research on Priority Areas 2002–2007 from The Ministry of Education, Culture, Sports, Science and Technology (Japan). Kougakutosho Ltd., Tokyo, 278 pp.

[B51] Mzighani SI Nikaido M Takeda M Seehausen O Budeba YL Ngatunga BP Katunzi EFB Aibara M Mizoiri S Sato T Tachida H Okada N (2010) Genetic variation and demographic history of the *Haplochromis laparogramma* group of Lake Victoria. An analysis based on SINEs and mitochondrial DNA. Gene 450: 39-47. doi: 10.1016/j.gene.2009.10.002 19837145

[B52] Niemantsverdriet P (2005) Victoriacichliden houden en kweken in het laboratorium. Cichlidae 31: 150-156.

[B53] Ogutu-Ohwayo R (1990) The decline of the native fishes of Lakes Victoria and Kyoga (East Africa) and the impact of introduced species, especially the Nile perch, *Lates niloticus* and the Nile tilapia, *Oreochromis niloticus*. Environmental Biology of Fishes 27: 81-96. doi: 10.1007/BF00001938

[B54] Rinkes M (1999) Conflicts in cichlid head morphology due to optimization of functional demands on suction feeding. PhD Thesis, Leiden, the Netherlands: Leiden University.

[B55] Rosenberger AE Chapman LJ (2000) Respiratory characters of three species of haplochromine cichlids: Implications for use of wetland refugia. Journal of Fish Biology 57: 483-501. doi: 10.1111/j.1095-8649.2000.tb02187.x

[B56] Seehausen O (1995) Cichliden von der tansanischen Victoriasee-Küste. In: Stawikowski, R (Ed). Cichliden, Festschrift zum 25jährigen Jubiläum der DCG. Deutsche Cichliden-Gesellschaft, Frankfurt am Main: 143-146.

[B57] Seehausen O (1996) Lake Victoria Rock Cichlids: Taxonomy, Ecology and Distribution. Verduijn Cichlids, Zevenhuizen, 304 pp.

[B58] Seehausen O Bouton N (1996) Polychromatism in rock dwelling Lake Victoria cichlids: types, distribution, and observations on their genetics. In: Konings A. (Ed) The Cichlids Yearbook, volume 6. Cichlid Press, 36–45.

[B59] Seehausen O Lippitsch E Bouton N Zwennes H (1998) Mbipi, the rock-dwelling cichlids of Lake Victoria: description of three new genera and fifteen new species (Teleostei). Ichthyological Explorations of Freshwaters 9: 129-228.

[B60] Seehausen O van Alphen JJM (1998) The effect of male colouration on female mate choice in closely related Lake Victoria cichlids (*Haplochromis nyererei* complex). Behavioural Ecology and Sociobiology 42: 1–8. doiL 10.1007/s002650050405

[B61] Seehausen O van Alphen JJM Lande R (1999) Color polymorphism and sex ratio distortion in a cichlid fish as an incipient stage in sympatric speciation by sexual selection. Ecology Letters 2: 367-378.

[B62] Seehausen O van Alphen JJM Witte F (1997a) Cichlid fish diversity threatened by eutrophication that curbs sexual selection. Science 277: 1808-1811. doi: 10.1126/science.277.5333.1808

[B63] Seehausen O van Alphen JJM Witte F (2003) Implications of eutrophication for fish vision, behavioral ecology and species coexistence: A theoretical framework. In: Crisman TL Chapman LJ Chapman CA Kaufman LS (Eds). Conservation, Ecology, and Management of African Fresh Waters. University Press of Florida, Florida: 268-287.

[B64] Seehausen O Witte F (1995) Extinction of many and survival of some: the current situation of the endemic cichlids in southern Lake Victoria. Tropical Fish Hobbyist 43 (7): 96-105.

[B65] Seehausen O Witte F Katunzi EF Smits J Bouton N (1997b)Patterns of the remnant cichlid fauna in southern Lake Victoria. Conservation Biology 11: 890-904. doi: 10.1046/j.1523-1739.1997.95346.x

[B66] Smit SA Anker GCh (1997) Photopic sensitivity to red and blue light related to retinal differences in two zooplanktivorous haplochromine species (Teleostei, Cichlidae). Netherlands Journal of Zoology 47: 9-20. doi: 10.1163/156854297X00201

[B67] Smith C Wootton RJ (1994) The cost of parental care in *Haplochromis* ‘argens’ (Cichlidae). Environmental Biology of Fishes 40: 99-104. doi: 10.1007/BF00002184

[B68] Smith C Wootton RJ (1995) The costs of parental care in teleost fishes. Reviews in Fish Biology and Fisheries 5: 7-22. doi: 10.1007/BF01103363

[B69] Snoeks J (1994) The haplochromines (Teleostei, Cichlidae) of Lake Kivu (East Africa). A taxonomic revision with notes on their ecology. Annalen van het Koninklijk Museum voor Midden-Afrika, Zoologische Wetenschappen 270: 1-221.

[B70] Tacon P Baroiller JF Le Bail PY Prunet P Jalabert B (2000) Effect of egg deprivation on sex steroids, gonadotropin, prolactin, and growth hormone profiles during the reproductive cycle of the mouthbrooding cichlid fish *Oreochromis niloticus*. General and Comparative Endocrinology 117: 54-65. doi: 10.1006/gcen.1999.7388 10620423

[B71] van der Meer HJ (1989) Ecological significance of retinal receptor patterns in 4 sympatric haplochromine cichlids. Annalenvan het Koninklijk Museum voor Midden-Afrika, Zoologische Wetenschappen 257: 51-56.

[B72] van der Meer HJ (1991a) Determination of photopic thresholds in two sympatric cichlids using optomotor response. Annalenvan het Koninklijk Museum voor Midden-Afrika, Zoologische Wetenschappen 263: 91-96.

[B73] van der Meer HJ (1991b) Ecomorphology of photoreception in haplochromine cichlid fishes. PhD Thesis, Leiden, the Netherlands: Leiden University.

[B74] van der Meer HJ (1995) Visual resolution during growth in cichlid fish: A morphological and behavioural case study. Brain, Behaviour and Evolution 45: 25-33. doi: 10.1159/000113383 7866769

[B75] van der Meer HJ Bowmaker JK (1995) Interspecific variation of photoreceptors in four co-existing haplochromine cichlid fishes. Brain, Behaviour and Evolution 45: 232-240. doi: 10.1159/000113552 7620872

[B76] van der Meer HJ Anker GCh Barel CDN (1995) Ecomorphology of retinal structures in zooplanktivorous haplochromine cichlids (Pisces) from Lake Victoria. Environmental Biology of Fishes 44: 115-132. doi: 10.1007/BF00005910

[B77] van der Meer HJ van Rijssel JC Wagenaar LC Witte F (2012) Photopic adaptations to a changing environment in two Lake Victoria cichlids. Biological Journal of the Linnean Society 106: 328-341. doi: 10.1111/j.1095-8312.2012.01859.x

[B78] van Oijen MJP Witte F (1996) Taxonomical and ecological description of a species complex of zooplanktivorous and insectivorous cichlids from Lake Victoria. Zoologische Verhandelingen Leiden 302: 1-56.

[B79] van Oijen MJP (1996) The generic classification of the haplochromine cichlids of Lake Victoria, East Africa. Zoologische Verhandelingen Leiden 302: 57–110. http://www.repository.naturalis.nl/document/148949

[B80] van Rijssel JC Witte F (2012) Adaptive responses in resurgent Lake Victoria cichlids over the past 30 years. Evolutionary Ecology. doi: 10.1007/s10682-012-9596-9

[B81] van Staaden MJ Huber R Kaufman LS Liem KF (1995) Brain evolution in cichlids of the African Great Lakes: brain and body size, general patterns, and evolutionary trends. Zoology 98: 165-178.

[B82] Verheijen E van der Linden A-M Decleir W (1989) The eye-lens proteins of haplochromine cichlids from Lake Victoria studied by isoelectric focusing. Annalenvan het Koninklijk Museum voor Midden-Afrika, Zoologische Wetenschappen 257: 93-96.

[B83] Wanink JH (1998) The pelagic cyprinid *Rastrineobola argentea* as a crucial link in the disrupted ecosystem of Lake Victoria. PhD Thesis, Leiden, the Netherlands: Leiden University.

[B84] Wanink JH Goldschmidt T Witte F (1989) Recent changes in the zooplanktivorous-/insectivorous fish community from the Mwanza Gulf of Lake Victoria. In: Anonymous (Eds). WOTRO Report for the year 1988. Netherlands Foundation for the Advancement of Tropical Research, The Hague, the Netherlands: 22-28.

[B85] Wanink JH Kashindye JJ Goudswaard PC Witte F (2001) Dwelling at the oxycline: does increased stratification provide a refugium for the Lake Victoria sardine *Rastrineobola argentea*? Freshwater Biology 46: 75–85. doi: 10.1111/j.1365-2427.2001.00644.x

[B86] Wanink JH Witte F (1998) Niche shift in a zooplanktivorous cyprinid from Lake Victoria after reduction of its cichlid guild members by Nile perch. In: Wanink JH (1998) The pelagic cyprinid *Rastrineobola argentea* as a crucial link in the disrupted ecosystem of Lake Victoria. PhD Thesis, Leiden, the Netherlands: Leiden University, 157–167.

[B87] Wanink JH Witte F (2000) The use of perturbation as a natural experiment: effects of predator introduction on the community structure of zooplanktivorous fish in Lake Victoria. Advances in Ecological Research 31: 553-570. doi: 10.1016/S0065-2504(00)31030-3

[B88] Witte F (1984a) Consistency and functional significance of morphological differences between wild-caught and domestic *Haplochromis squamipinnis* (Pisces, Ciclidae). Netherlands Journal of Zoology 34: 596-612. doi: 10.1163/002829684X00308

[B89] Witte F (1984b) Ecological differentiation in Lake Victoria haplochromines: comparison of cichlid species flocks in African Lakes. In: Echelle AA Kornfield I (Eds). , Evolution of Fish Species Flocks. Orono Press, University of Maine, Maine: 155-167.

[B90] Witte F (1987) From form to fishery. An ecological and taxonomical contribution to morphology and fishery of Lake Victoria cichlids. PhD Thesis, Leiden, the Netherlands: Leiden University.

[B91] Witte F Barel CDN van Oijen MJP (1997) Intraspecific variation of haplochromine cichlids from Lake Victoria and its taxonomic implications. South African Journal of Science 93: 585–594.

[B92] Witte F Goldschmidt T Goudswaard PC Ligtvoet W van Oijen MJP Wanink JH (1992a) Species extinction and concomitant ecological changes in Lake Victoria. Netherlands Journal of Zoology 42: 214-232.

[B93] Witte F Goldschmidt T Wanink J van Oijen M Goudswaard K Witte-Maas E Bouton N (1992b) The destruction of an endemic species flock: quantitative data on the decline of the haplochromine cichlids of Lake Victoria. Environmental Biology of Fishes 34: 1-28. doi: 10.1007/BF00004782

[B94] Witte F van der Meer H Barel K (2003) Door cichlidenogen bezien: het belang van het gezichtsvermogen bij haplochromine cichliden voor het ontstaan en in stand houden van diversiteit. Cichlidae 29: 104-114.

[B95] Witte F Msuku BS Wanink JH Seehausen O Katunzi EFB Goudswaard PC Goldschmidt T (2000) Recovery of cichlid species in Lake Victoria: an examination of factors leading to differential extinction. Reviews in Fish Biology and Fisheries 10: 233-241. doi: 10.1023/A:1016677515930

[B96] Witte F Seehausen O Wanink JH Kishe-Machumu M Rensing M Goldschmidt T (2012a) Cichlid species diversity in naturally and anthropogenically turbid habitats of Lake Victoria, East Africa. Aquatic Sciences 74. doi: 10.1007/s00027-012-0265-4

[B97] Witte F Silsbe GM Hecky RE Goudswaard PC Guildford SJ Kishe-Machumu MA Wanink JH (2012b) Did the loss of phytoplanktivorous fish contribute to algal blooms in the Mwanza Gulf of Lake Victoria? Hydrobiologia 679: 283–296. doi: 10.1007/s10750-011-0893-z

[B98] Witte F Wanink JH (2000) De zoöplanktivore cichliden van het Victoriameer: ontdekking, verdwijning en terugkeer. Cichlidae 26: 77-84.

[B99] Witte F Wanink JH Kishe-Machumu M (2007a) Species distinction and the biodiversity crisis in Lake Victoria. Transactions of the American Fisheries Society 136: 1146-1159. doi: 10.1577/T05-179.1

[B100] Witte F Wanink JH Kishe-Machumu M Mkumbo OC Goudswaard PC Seehausen O (2007b) Differential decline and recovery of haplochromine trophic groups in the Mwanza Gulf of Lake Victoria. Aquatic Ecosystem Health and Management 10: 416-433. doi: 10.1080/14634980701709410

[B101] Witte F Wanink JH Rutjes HA van der Meer HJ van den Thillart GEEJM (2005) Eutrophication and its influences on the fish fauna of Lake Victoria. In: Reddy VM (Ed). Restoration and Management of Tropical Eutrophic Lakes. Science publishers, Inc., Enfield (NH): 301-338.

[B102] Witte F Welten M Heemskerk M van der Stap I Ham L Rutjes C Wanink J (2008) Major morphological changes in a Lake Victoria fish within two decades. Biological Journal of the Linnean Society 94: 41-52. doi: 10.1111/j.1095-8312.2008.00971.x

[B103] Witte F Witte-Maas ELM (1981) Haplochromine cleaner fishes: a taxonomic and eco-morphological description of two new species. Netherlands Journal of Zoology 31: 203-231. doi: 10.1163/002829680X00249

[B104] Witte F Witte-Maas ELM (1987) Implications for taxonomy and functional morphology of intraspecific variation in haplochromine cichlids of Lake Victoria. In: Witte F, From form to fishery, PhD Thesis, Leiden, the Netherlands: Leiden University. 1–83.

[B105] Zihler F (1982) Gross morphology and configuration of the digestive tract of Cichlidae (Teleostei, Perciformes): phylogenetic and functional significance. Netherlands Journal of Zoology 32: 544-571. doi: 10.1163/002829682X00210

